# Strong and Anomalous Thermal Expansion Precedes the Thermosalient Effect in Dynamic Molecular Crystals

**DOI:** 10.1038/srep29610

**Published:** 2016-07-12

**Authors:** Manas K. Panda, Roberto Centore, Mauro Causà, Angela Tuzi, Fabio Borbone, Panče Naumov

**Affiliations:** 1 New York University Abu Dhabi, PO Box 129188, Abu Dhabi, United Arab Emirates; 2 University of Naples Federico II, Department of Chemical Sciences, Via Cintia, I-80126, Naples, Italy; 3 University of Naples Federico II, Department of Chemical, Materials and Production Engineering, Piazzale V. Tecchio, I-80125, Naples, Italy

## Abstract

The ability of thermosalient solids, organic analogues of inorganic martensites, to move by rapid mechanical reconfiguration or ballistic event remains visually appealing and potentially useful, yet mechanistically elusive phenomenon. Here, with a material that undergoes both thermosalient and non-thermosalient phase transitions, we demonstrate that the thermosalient effect is preceded by anomalous thermal expansion of the unit cell. The crystal explosion occurs as sudden release of the latent strain accumulated during the anisotropic, exceedingly strong expansion of the unit cell with *α*_a_ = 225.9 × 10^−6^ K^−1^, *α*_b_ = 238.8 × 10^−6^ K^−1^ and *α*_c_ = −290.0 × 10^−6^ K^−1^, the latter being the largest negative thermal expansivity observed for an organic compound thus far. The results point out to the occurence of the thermosalient effect in phase transitions as means to identify new molecular materials with strong positive and/or negative thermal expansion which prior to this work could only be discovered serendipitously.

Plants, insects and animals have developed fascinating mechanisms for passive or active motility required for dispersal, defense and survival^1^,^2^,^3^,^4^,^5^,^6^,^7^,^8^,^9^,^10^. In 2010, we reported in amazement that in distant analogy with rapidly motile biological systems, when excited by heat or light certain molecular crystals are able to launch and propel themselves, travelling over large distances without chemical reaction/decomposition^11^. These *thermosalient* (TS) and *photosalient* (PS) effects^12^,^13^,^14^,^15^,^16^ are mechanistically impressive phenomena which demonstrate that dynamic molecular crystals can accumulate extraordinarily high elastic energy—a property that if controlled could be put to a good use within the realm of alternative transduction of thermal energy into mechanical work. Self-actuation of a still object, such as a crystal, requires that it navigates three challenges: (1) generation of stress by a phase transition or chemical reaction, (2) accumulation of elastic energy over long range, and (3) sudden release of the accumulated energy by rapid mechanical reconfiguration or ejection of debris. Typically, the TS and PS crystals reach an in-plane component of the speed of 0.2–0.8 m s^−1^ and carry kinetic energy on the order of 1–10^4^ pJ^17^. The times to reach these velocities are incredibly short, with the debris being able to accelerate itself in less than a millisecond. The crystals or the debris travel distances that normally range from several millimeters to several centimeters (longer distances are anticipated in absence of drag from the air). The current record-holder in respect to the distance that crystals of TS materials can reach is *N*′-2-propylydene-4-hydroxybenzohydrazide, an imine of acetone (IMACET, Fig. 1) whose crystals were recently reported by some of us to be able to travel distances of up to one meter in air^18^. The mechanical effect in these crystals can be stimulated by a stochastic event or by applying localized external pressure to trigger the phase transition. The accumulated strain energy is accrued until the point when an instantaneous release is triggered that results in crystal explosion or hopping (Fig. 2). The strain energy is partitioned between the elastic energy of disintegration that accounts for crystal fracture and the kinetic energy that is carried away by the fragments that fly off. However, understanding of the structural subtleties that are specific to the exotic properties of these materials remains elusive. Here we report that the thermosalient effect in IMACET is preceded by anomalous distortion of the unit cell with remarkably strong biaxial *positive* expansion. The material also exhibits the largest uniaxial *negative* expansions reported thus far. To study the effects of the strength of intermolecular interactions and the molecular mass on the the thermosalient effect, two deuterated variants of IMACET were also prepared. When taken together with the previous studies, the results indicate that the occurence of the thermosalient effect could be used to identify solids with anomalous thermal behavior, including new materials with large negative expansion that are of essence to organic electronics. It is also concluded that the temperatures of the thermosalient transitions are sensitive to and can be altered by deuteration.

## Results and Discussion

IMACET was prepared according to a published procedure^18^ and its deuterated analogues were obtained by using deuterated precursors and characterized by ^1^H NMR spectroscopy (Supplementary Figures 1–4) and variable-temperature (VT) IR spectroscopy (Supplementary Figure 5). The thermal behavior of the polymorphs was characterized by differential scanning calorimetry (DSC; Fig. 3 and Supplementary Figure 6) and VT powder X-ray diffraction (Supplementary Figures 7 and 8). To obtain insight into the role of the intermolecular interactions on the TS transition, two isotopically labeled variants were also prepared and characterized—IMACET-D_2_, deuterated in the acidic protons, and IMACET-D_6_, deuterated in the methyl groups (typical habits obtained by recrystallization are shown in Supplementary Figure 9). The phase transitions were monitored by optical microscopy. Wherever possible, native crystals were used to avoid suppression of the effect by grinding^17^.

**Figure 2:**
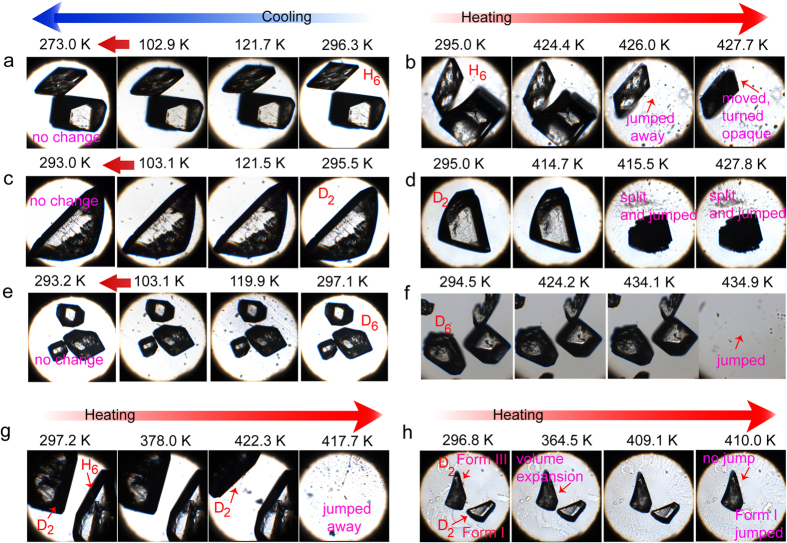
Thermal behavior and thermosalient effect in crystals of protiated and deuterated variants of IMACET recorded by variable-temperature optical microscopy. (**a**) IMACET-H_6_ on cooling by liquid nitrogen. (**b**) IMACET on heating. (**c**) IMACET-D_2_ on cooling. (**d**) IMACET-D_2_ on heating. (**e**) IMACET-D_6_ on cooling. (**f**) IMACET-D_6_ on heating (the background of this set of images appears darker due to the use of polarizing filter). (**g**) IMACET and IMACET-D_2_ on heating. (**h**) Form I and form III of IMACET-D_2_ on heating.

IMACET has been classified as one of the only few materials that belong to the class III TS solids^14^,^17^—crystalline solids that are distinct from the other two classes by having networks of low-dimensional hydrogen bonds that can accumulate elastic strain due to molecular distortion thereby effectively temporarily suspending a structural phase transition. It can be switched thermally between three orthorhombic phases (forms I, II and III) of which only the irreversible transition from form I to form II is accompanied by a TS effect^17^,^18^. As shown with the images in Fig. 2, on heating all crystals undergo irreversible phase transition from form I to form II. The transition I → II is accompanied by a strong TS effect whereby the crystals fiercely explode and hop off the stage (Supplementary Movies 1–5). The deuterated crystals are also TS-active on heating (Supplementary Movies 6–10). These events are accompanied by endothermic effects and a saw-tooth profile in the differential scanning calorimetry (DSC) measurements—a signature of other TS solids^17^ and inorganic martensitic phases^19^,^20^.

**Figure 3:**
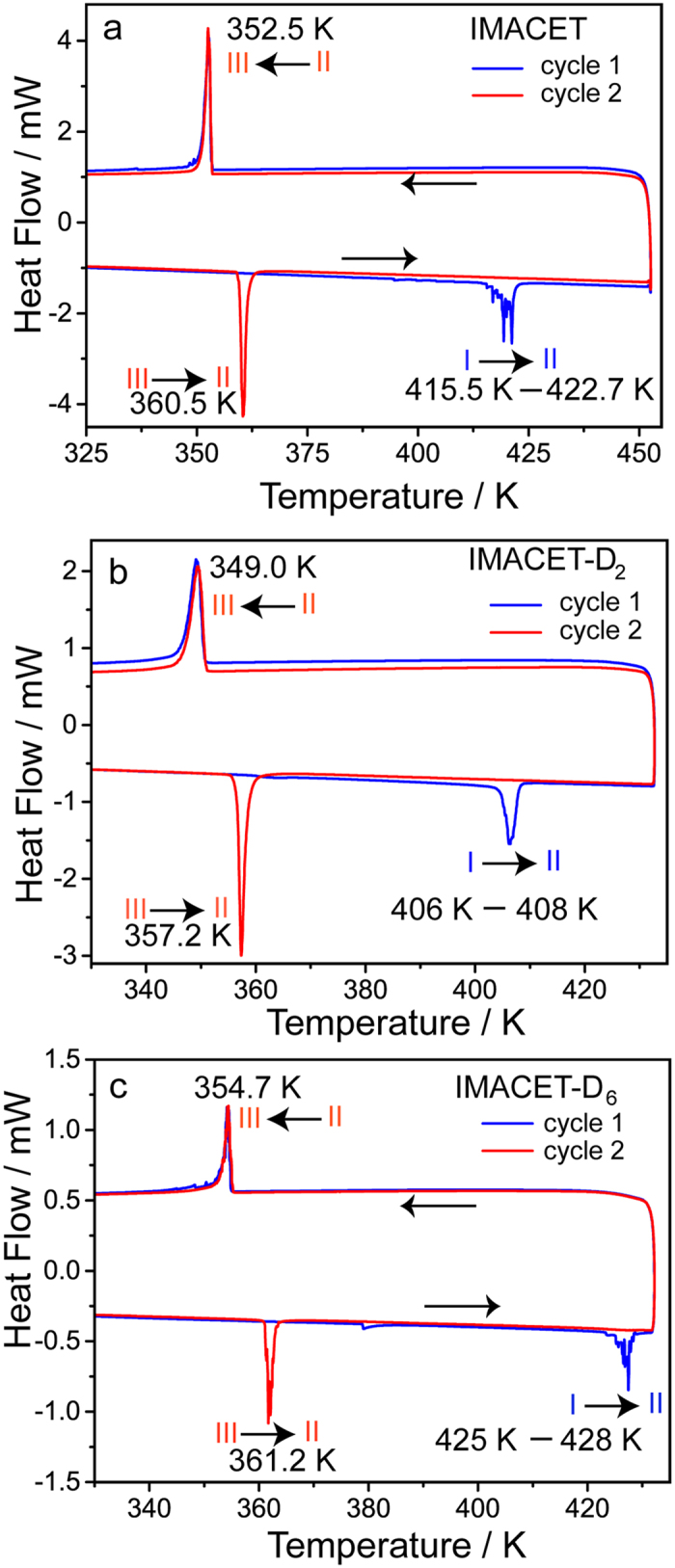
Thermal (DSC) profile of crystals of IMACET, IMACET-D_2_ and IMACET-D_6_ above room temperature showing the phase transitions between forms I–III. The two consecutive thermal cycles are shown in different colors.

The thermal (DSC) profile of the phase transitions of as-synthesized, unground crystals of IMACET, IMACET-D_6_ and IMACET-D_2_ show that the temperature of transition I → II is affected by deuteration (Fig. 3). Deuteration at the methyl groups (IMACET-D_6_) *increases* (425–428 K) while deuteration of the NH/OH groups (IMACET-D_2_) *decreases* (406–408 K) the transition temperature relative to the protiated (non-deuterated) compound (415.5–422.7 K). The opposite trend observed with the two isotopomers could indicate that the isotope effect on transition temperature is not a mere consequence of different crystal density related to the molecular mass, where retardation of the transition (shift to higher temperature) could be expected for both deuterated variants. Instead, the isotopic substitution appears to affect the transition by altering the hydrogen bonding strength (see below). This result highlights the role of the intermolecular interactions on the evolution and progression of the elastic waves that drive the TS effect. On cooling from 450 K all crystals of thermally generated form II undergo non-TS transition II → III, which is accompanied by exothermic effect at 353, 354 and 349 K for IMACET, IMACET-D_6_ and IMACET-D_2_, respectively. As shown with Supplementary Figure 6 and Supplementary Movies 11–13, no thermal effects could be detected below room temperature (down to 103 K). Unlike the irreversible transition I → II, in all deuterated variants the transition II ↔ III is reversible and can be repeated by heating-cooling cycles (Fig. 3). As a future prospect, one could control the temperature of mechanical response by using mixtures of deuterated and non-deuterated samples. Such temperature tuning of the effect could be relevant for applications of TS materials in energy transduction devices.

**Figure 4:**
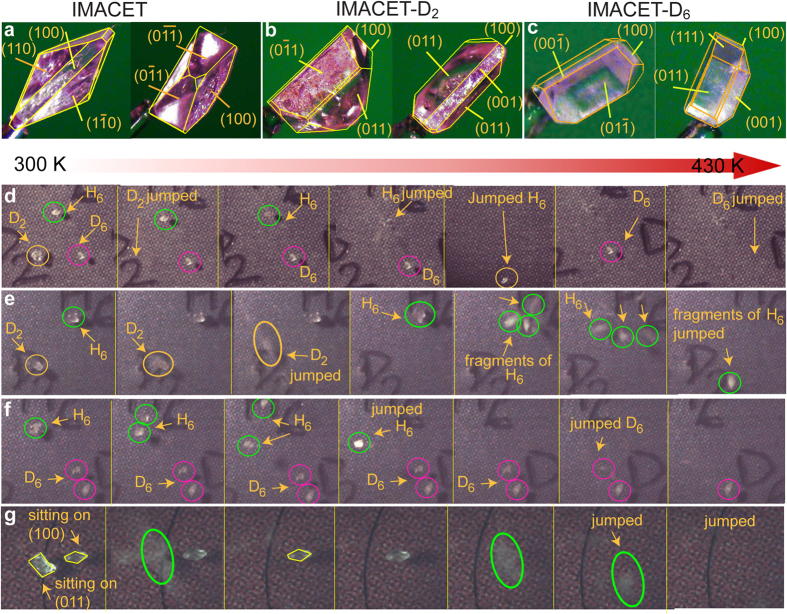
Face indices (**a–c**) and snapshots of the thermosalient effect in crystals of the three isotopic variants of IMACET (**d–g**). The images of protiated IMACET (denoted H_6_ for clarity; green circles), IMACET-D_2_ (D_2_, yellow circles) and IMACET-D_6_ (D_6_, purple circles) in panels (d–g) were extracted from high-speed video recordings (recording rate: (2-5)·10^3^ s^−1^).

The rapid motion of IMACET crystals was observed with a microscope coupled to a hot stage and recorded with high-speed camera (frame rate (2–5)·10^3^ s^−1^; Supplementary Movies 2–5). The kinematic analysis revealed that the motion is not uniform and occurs as a variety of kinematic effects ranging from small displacements to bursting to fierce hopping off the stage (Fig. 4d–g). For direct comparative assessment of the kinematics of deuterated and non-deuterated crystals and to account for eventual size effects, several batches of crystals of similar size were hand-picked and simultaneously heated. In line with the order of transition temperatures determined by DSC (Fig. 3), on heating to high temperature, the time required for stress accrual before the crystals hopped increases in the order IMACET-D_2_ < IMACET < IMACET-D_6_ (Fig. 4d–f; Supplementary Movies 14–16). This result confirmed that by affecting the TS transition temperature, the deuteration has strong effect on crystal motility. The contrasting effect on the transition temperature by D_2_- and D_6_-deuteration can be interpreted as a tradeoff between two factors, the strength of intermolecular interactions and molecular weight. The retardation of the phase transition by deuteration in the methyl positions (D_6_) is related to increase in molecular mass. Deuteration of the N/O protons (D_2_), on the other hand, brings about small increase in molecular mass, however the effect is outweighed by strengthening of the hydrogen bonding^21^,^22^,^23^,^24^. This conclusion parallels the key role that the intermolecular interactions play in the evolution of the mechanical effect of class III TS solids, in contrast with class I and class II. Due to difficulties with maintaining the optical focus on the crystals during the experiment, it was not possible to distinguish between the most common types of motion (kinematic patterns) of protiated and deuterated crystals.

The anisotropic volume change of the crystals during the phase transition, which ultimately drives the TS effect, can be considered a result of differential growth of the crystal faces of form I upon its conversion to form II. A thermodynamic approach to understand this anisotropy is to calculate the surface formation energies within the Wulff’s theory that describes the thermodynamic equilibrium shape of the crystal^25^. Within this theorem, the equilibrium shape is considered a result of planes parallel to the faces whose distance from the barycenter is proportional to the surface formation energy.

The crystals of the three forms of IMACET and selected surfaces were modeled by employing the slab approach (for details of the calculations, see the Supplementary Methods). Thin films (2D periodic systems) parallel to the (*hkl*) planes that define the surfaces were sliced out from a previously optimized bulk structure. Being a wide band gap molecular insulator, the slab included only complete molecules. The properties rapidly converged with thickness (the convergence of the surface formation energy with slab thickness was confirmed). Depending on the spacing of the (*hkl*) lattice planes, the slab thickness necessary for convergence (based on the similarity with the bulk-like behavior in the middle of the slab) was in the range 1.5–2.0 nm. The surface formation energy (surface tension) *γ* was calculated^26^ for each crystal face of each crystallographic phase as *γ* = (*E*_slab_−*E*_bulk_)/2*nA*, where *E*_slab_ is the total energy of the slab model, *E*_bulk_ is the energy of the bulk, *n* is the number of bulk unit cells present in the converged slab, and *A* is the area of the 2D unit cell in the slab (the factor 2 accounts for the two surfaces parallel to the given crystal face within the slab model).

**Figure 5:**
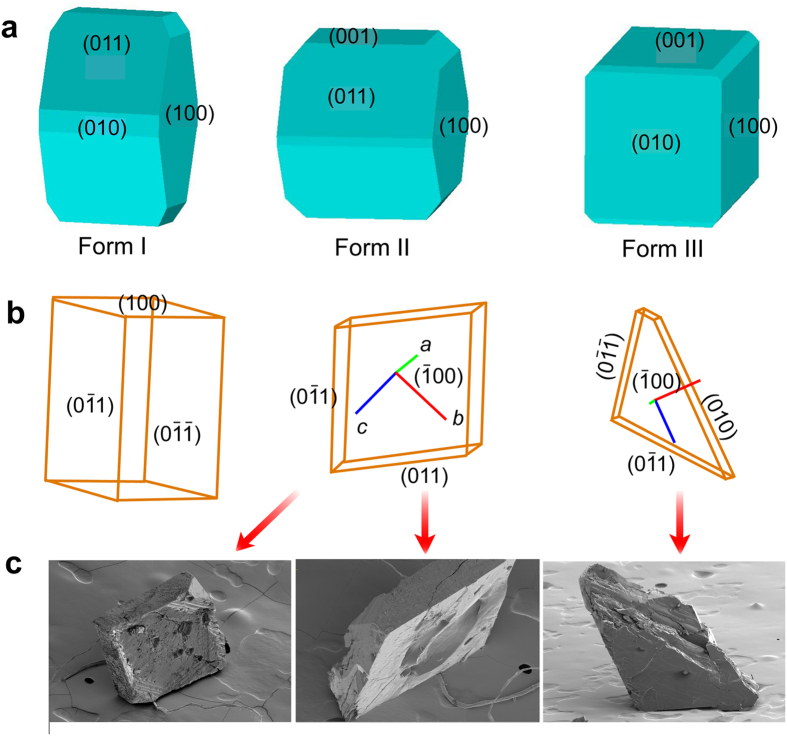
Correlation among surface tension, crystal habit and modes of parting of freely standing IMACET crystals during the thermosalient transition. (**a**) Wulff-Gibbs polyhedra calculated for forms I, II and III. (**b**) Face indices of the crystal before the transition (left), and of two typical fragments obtained after the phase transition (center, right). (**c**) SEM images of the typical fragments, schematically shown in panel b, that are obtained by splintering of the crystals in the course of the thermosalient transition (the first and the second image show different views of the same fragment).

The results from the calculations of the surface tension and relative area of the main crystal faces for the three orthorhombic polymorphs of IMACET are reported in Table 1, and the crystal models are depicted as Wulff-Gibbs polyhedra in Fig. 5a. In crystals of forms I and II, the widest faces are (100) and (011) and their Friedel/symmetry counterparts, (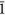
00) and (0
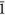

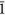
). In line with the structural similarity between forms I and II inferred from the cell similarity index^17^, these two forms have very similar surface energies. The calculated surface tension conforms to the morphology observed with crystals of phase I which appear as elongated prisms with well developed (011), (0
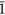
1), (100) and (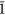
00) faces (note that indexing of the smaller crystal faces is irrelevant for the purpose of this study, and was not attempted). The calculated changes during the non-TS transition from form II to form III are much more pronounced; this transition is accompanied by expansion on (001) and (010) and shrinking on (011). We note that based strictly on morphology the mode of parting of crystals of form I during the TS transition appears to be related with the pattern of the calculated surface tensions. The new surfaces created upon splintering correspond to crystal faces with low surface tension, as reported in Table 1 (i.e., (100) and (010) for phase III).

In several experiments, free-lying individual single crystals of form I were taken in turn over the TS transition by heating on a hot plate, whereby they sprang off and/or splintered. Any large fragments that were obtained in addition to the smaller debris were collected, face-indexed by optical microscopy and examined by scanning electron microscopy (SEM). The one-to-one correspondence of the unit cells of the forms I and II^17^,^18^ facilitated structure comparison.

As a typical crystal habit, Fig. 5b shows schematic representation of a 1.5 mm-thick prismatic crystal with lozenge-shaped bases. The crystallographic axes *b* and *c* are parallel to the diagonals of the lozenge faces, while the *a* axis runs along the long side of the prism. After the TS transition, two large pieces were retrieved from the debris, which are also sketched in Fig. 5b. One of the fragments had faces (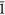
00) and (100) with comparable area to the parent crystal, however it was only 0.16 mm thick along *a*. Thus, this fragment had separated out from the parent crystal by parting along the planes (100) or (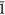
00). Similar to the first fragment, the second fragment also had wide (100) and (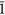
00) faces, but was thick 0.3 mm along *a*. Comparison of the crystal habits of the two fragments (Fig. 5b) indicates that the second fragment was obtained from a fragment similar to the first fragment that additionally split along (010) and lost a piece (the complementary piece was recovered from the debris). These two habits of the crystal debris were fairly consistent across multiple experiments, indicating that crystals typically disintegrate by splitting normal to the crystallographic axis *a*, and in some cases by splitting normal to the *b* axis. Inspection of the fragments by SEM (Fig. 5c) showed relatively smooth surface along the new faces (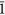
00) and (100). On the other hand, the newly generated faces (010) were uneven and had visible indentations, ditches and other defects. Thus, while the crystals split cleanly perpendicular to the *a* axis, splitting perpendicular to the *b* axis results in rugged surface.

**Figure 6:**
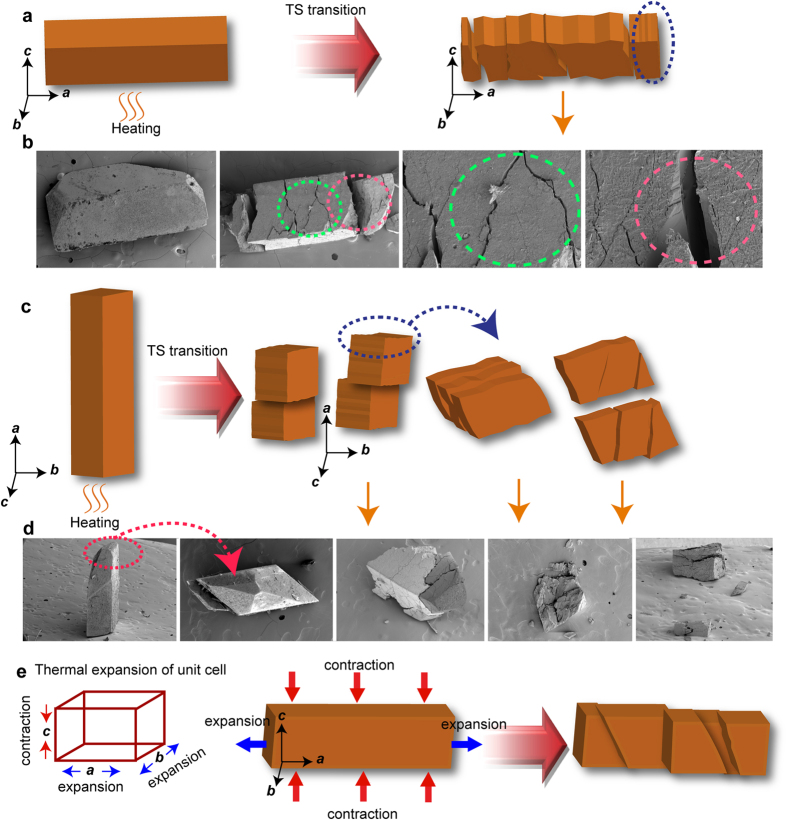
Correlation between the mode of splitting, crystal habit and thermal expansion during the thermosalient effect of affixed (constrained) IMACET crystals. (**a**) Schematic of a typical disintegration of a crystal glued on the (0
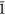
1) face. (**b**) SEM images of a typical crystal glued with its (0
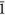
1) face before (far left panel) and after the phase transition (the other panels). The circled areas are zoomed for clarity. (**c**) Schematic showing typical disintegration of a crystal that was glued with its (100) face onto the basis (**d**) SEM images before (the two panels on the far left) and after (the other panels) the transition of a typical crystal glued onto the surface with its (100) face. The dashed red arrow shows top view of the parent crystal before the transition. (**e**) Correlation between the mode of splitting of the crystal and the thermal expansion that causes build-up of stress.

Fixation of the crystals to solid support by using minimal amount of glue restrains their deformation prior to explosion and results in different kinematic outcome (Fig. 6). When the crystals are affixed with their large lateral faces (011) or (01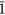), the TS transition usually results in transverse cracks and fissures, with minor parting of the fragments (Fig. 6a,b). Instead, when the crystals are affixed with their basal face (100) or (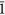
00), they explode into fragments of variable size (Fig. 6c,d). The basal part of the crystals remains attached, while the TS transition induces vertical propulsion of the leaving fragments that were found scattered over the microscope stage. Similar to free (unconstrained) specimens, these crystals split with a clean surface perpendicular to the *a* axis.

**Figure 7:**
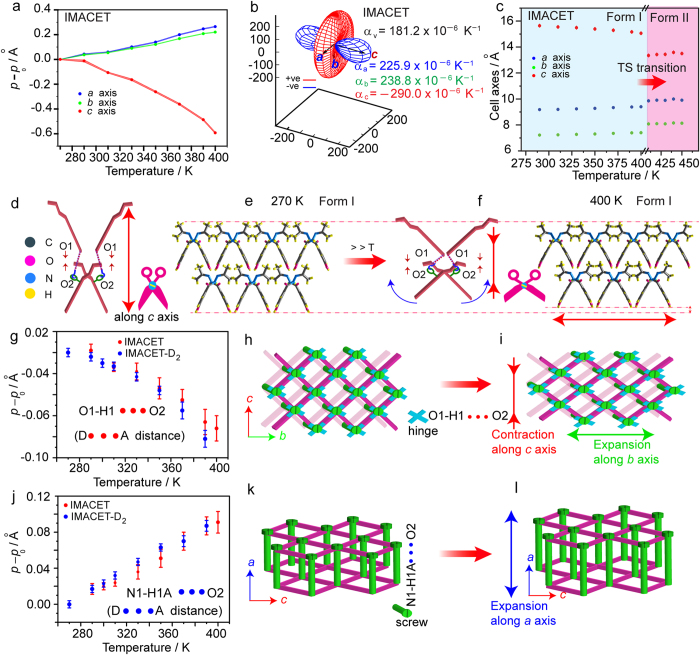
Thermal expansion and mechanism of the TS effect in IMACET. (**a**) Thermal variation of the unit cell axes (the standard deviations are shown as the thickness of the connecting lines). (**b**) Thermal expansion coefficients and plot of the expansivity indicatrices along the principal axes calculated as *α*_*l*_  = (1/*l*) (δ*l*/δ*t*)_*P*_ with PASCal^27^. The principal axes X_1_, X_2_ and X_3_ coincide with the crystallographic axes *c*, *a* and *b*, respectively. Red and blue colors represent positive and negative thermal expansion. (**c**) Change of the unit cell parameters across the TS phase transition (the standard deviations are smaller than the symbols). (**d–f**) Schematic representation of the intermolecular hydrogen bond O1—H1···O2 related to the negative thermal expansion along the *c* axis. (**g,j**) Variations of the distance between the donor (D) and the acceptor (A) in the hydrogen bonds O1—H1···O2 and N1—H1A···O2 that account for anisotropy in the thermal expansion. (**h,i**) Cartoon showing gradual decrease in the O1···O2 distance on heating that results in supramolecular jack-like distortion. This deformation causes concomitant positive thermal expansion along the *b* axis and negative thermal expansion along the *c* axis. (**k,l**) Cartoon showing the increase in the distance of the hydrogen bond N1···O2 that acts as hinge-screw. The elongation of this bond on heating accounts for the positive thermal expansion along the *a* axis.

Most of the TS phase transitions are isosymmetric, that is, the crystal group symmetry is preserved, and in many cases the changes in the unit cell volume as well as the structural changes at a molecular level are unexceptional^14^,^16^,^17^. Based on the preceding discussion, it can be concluded that the mode of splintering of IMACET is related to changes in the unit cell that occur before and during the transition. The fortuitous one-to-one correspondence between the unit cell parameters of forms I and II of IMACET allows direct comparison of their packing structures. The transition of form I (298 K) to form II (373 K) is accompanied by strongly anisotropic deformation where two axes, *a* (+8.8%) and *b* (+12.3%) expand. As shown in Fig. 7a–c, however, axis *c contracts* (~−15%) on transitioning of form I to form II. These changes that occur during the phase transition can be considered in view of the thermal expansion of the crystals of form I preceding the phase transition, a process that “prepares” the structure for switching to that of form II. Indeed, inspection of the temperature profile of the unit cell preceding the transition provides a hint for the reasons behind the observations. The thermal expansion along all three orthogonal axes in form I of all three isotopic variants is anomalous, with strong positive thermal expansion (PTE) along *a* and *b* axes of *α*_*a*_ = 225.9 × 10^−6^ K^−1^ and *α*_*b*_ = 238.8 × 10^−6^ K^−1^, respectively, and negative thermal expansion along the *c* axis, *α*_*c*_ = −290.0 × 10^−6^ K^−1^ in case of the non-deuterated IMACET (Fig. 7a–c). These thermal expansion coefficients, calculated by using the equation *α*_*l*_ = (1/*l*)(δ*l*/δ*t*)_*P*_^27^ are higher than the values that are commonly considered normal for molecular solids (0–20 × 10^−6^ K^−1^) and are extraordinarily high for an organic material. To the best of our knowledge, the NTE of IMACET along the *c* axis, −290.0 × 10^−6^ K^−1^, is the highest NTE values recorded for an organic crystal. It also surpasses the high NTE values reported for organic and metal-containing compounds, Ag_3_[Co(CN)_6_] (−130 × 10^−6^ K^−1^)^28^,^29^, metal organic framework FMOF-1 (−170 × 10^−6^ K^−1^)^30^, (*S*,*S*)-octa-3,5-diyn-2,7-diol (−205 × 10^−6^ K^−1^)^31^, Cd(34pba)(44pba)], MCF-82 (−218 × 10^−6^ K^−1^)^32^, and [Zn(L)_2_(OH)_2_]_*n*_·*n*CH_3_OH (L = 4-(1*H*-naphtho[2,3-d]imidazol-1-yl)benzoic acid; −21 × 10^−6^ K^−1^)^33^. This value seconds only the negative expansion reported recently for the metal-organic framework copper(II) tricyanomethanide, −407(28) × 10^−6^ K^−1 ^34 (Note: An anonymous reviewer of this article has brought to the authors’ attention that this value was calculated over a temperature range where the expansion is nonlinear and therefore may not be directly comparable to the values reported here or to other reported values for colossal thermal expansion).

In another organic TS solid, the chiral L-pyroglutamic acid, one of the two forms (β) associated with the TS phase transition (in this case, the high-temperature form) undergoes biaxial NTE (*α*_*a*_ = −54.8(8) × 10^−6^ K^−1^, *α*_*c*_ = −3.62(8) × 10^−6^ K^−1^) and exceptionally large uniaxial thermal expansion (*α*_*b*_ = 303(1) × 10^−6^ K^−1^) (note that these values were obtained by fitting powder X-ray diffraction data with the same formula used in this work)^16^. In a palladium organometallic TS material, the thermal expansion coefficients of phase α that undergoes TS transition to form *γ* are *α*_*a*_ = 260.4(3) × 10^−6^ K^−1^, *α*_*b*_ = 39.4(4) × 10^−6^ K^−1^ and *α*_*c*_ = −79.9(2) × 10^−6^ K^−1^ (these values were calculated along the three (non-orthogonal) crystallographic unit cell axes by using the equation *p* = *p*_0_{1 + *α*_*p*_(*T*−*T*_0_)[1 + *α*′*p*(*T*−*T*_0_)]} from powder X-ray diffraction data)^12^. Together with the results on IMACET presented here, these results indicate that the anomalous thermal expansions is invariably associated with TS effect as a prerequisite for crystal motility and explosion. As shown by the expansive indicatrices and the thermal expansion coefficients in Fig. 7b and Supplementary Figure 10, the thermal expansion of the deuterated crystals follows identical trend with similar expansivities. The shapes of the expansion plots along different axes of IMACET and IMACET-D_2_ are also similar, however both are different from that of IMACET-D_6_, as expected from the differences in hydrogen bonding strength caused by H/D substitution^24^.

Comparison of single crystal data at 270 K and 400 K showed that variation of coordination framework arises from the anisotropic change in hydrogen bonding. The relevant hydrogen bonds are depicted in Supplementary Figure 11 (see also Supplementary Table 5). In a rather simplified representation of the crystal structure, the hydrogen bond between the amide nitrogen and carbonyl oxygen (N1—H1A···O2) acts like a “hinge screw”^32^ along the crystallographic *a* axis. These screws connect the molecules in the *bc* plane. As shown in the plots in Fig. 7j–l, this distance increases with increasing temperature (270–400 K) and accounts for the PTE along the *a* axis. The distance of the O1—H1···N2 hydrogen bond (roughly parallel to the *a* axis) also increases with increasing temperature and accounts for the PTE along the *a* axis (Supplementary Figure 12). On the contrary, increasing temperature brings about apparent *contraction* of the hydrogen bond O1—H1···O2 (Fig. 7g–i). This contraction causes shrinking of the unit cell and is responsible for the uniaxial NTE along the *c* axis. Since at a molecular level contraction of a hydrogen bond by heating is not viable, it is likely that this apparent “contraction” is in fact an effect of the expansion of a stronger hydrogen bond, that is, O1—H1···N2, where the bond rotates around the hydrogen atom H1. Supplementary Movie 17 contains stick models of the structure showing the changes that occur by heating of the crystal to the TS transition and during the transition. The anisotropic changes in the unit cell are reflected in “breathing” of the structure—reversible expansion and contraction of the crystal when it is thermally cycled over the reversible phase transition III ↔ II (Supplementary Movie 18). As shown in Fig. 7g,j, the uniaxial distortions along the unit cell axes are not mutually independent; the hydrogen bonding contraction along the *c* axis results in a jack-like deformation and expansion along the *b* axis, causing PTE in that direction (Fig. 7d–f, Supplementary Movie 17). The characteristics of the new faces that evolve upon parting of the crystals are in accord with this model of an anisotropic, yet flexible structure: while the amide-like chains predominate along the *a* axis, there are no hydrogen-bonded chains along the *b* axis.

## Conclusions

The TS effect provides visually impressive demonstration of the action of elastic energy that can be stored within the crystal lattice of ordered solid matter. Mechanistically, these dynamic processes are related to diffusionless, synchronous (“military”) transitions whereby the accrued energy is suddenly released—if attained below a critical value—into kinetic energy and drives crystal reshaping. Higher energies take the crystal out of the elastic regime and lead to development of cracks, causing disintegration. Here we demonstrate that the elastic strain can be modulated by isotopic substitution. More importantly, the TS transitions are preceded by exceedingly strong anomalous expansion of the unit cell. At a supramolecular level, these peculiarities are rooted in the thermal anisotropy of hydrogen bonding interactions. Crystals of *N*′-2-propylydene-4-hydroxybenzohydrazide (IMACET) studied here can travel distances of up to about one meter when the strain energy related to strong biaxial thermal expansion and uniaxial negative thermal expansion is released during a thermosalient phase transition. In the crystal of this material, the expansion of the hydrogen bonding network causes PTE along the *a* axis, whereas shortening of the distance between the hydroxyl oxygen and carbonyl oxygen causes NTE along the *c* axis and results in the largest negative expansion in an organic compound observed thus far. Within a broader perspective, the results indicate that the TS effect can have predictive value in identifying organic materials with negative thermal expansion which can be utilized as non-expanding organic electronic components.

## Methods

### Synthesis and deuteration

IMACET was synthesized following a published procedure^18^. The deuterated analogues (99% D) were prepared by using deuterated reactants and solvents (for details on the synthetic, crystallization and characterization procedures, see the Supplementary Methods).

### Optical microscopy

The thermal behavior was analyzed using an optical hot stage microscope composed of temperature-controlled stage THMS600-PS (Linkam) mounted on Q-imaging (Q32643) optical microscope. Samples were heated or cooled to specific temperature from room temperature (300 K) at a constant heating rate of 5 K min^−1^. The kinematic behavior was recorded with high-speed camera (HotShot 1280 CC, NAC) mounted on Stereozoom SMZ745T Trinocular Stereoscope (Nikon).

### Electron microscopy

FESEM FEI Nova NanoSEM 450 instrument was used to image crystals with Scanning Electron Microscopy (SEM). The crystals were attached to a carbon tape and sputtered in a DentonVacuum Desk V HP sputterer using an alloy of gold/palladium target prior to observation.

### X-ray diffraction (XRD) analysis

Single crystal XRD data were collected on a Bruker SMART APEX II diffractometer with monochromated Mo*K*_α_ radiation (*λ* = 0.71069 Å) equipped with CCD area detector. Data reduction was performed using the SAINT software and analyzed for agreement using XPREP^35^. Absorption correction was applied with SADABS^36^. All structures were determined by the methods included in the SHELXT program of the APEX software suite and refined using SHELXL-2014^37^,^38^,^39^,^40^. The non-hydrogen atoms were refined anisotropically. The hydrogen atoms bonded to oxygen and nitrogen atoms were located from the electron density map and refined by using the AFIX 147 and AFIX 43 commands. The aromatic and aliphatic hydrogens were treated using AFIX 43 and AFIX 137, respectively. For deuterated molecules, the deuterium atoms were assigned by changing the scattering factor of the hydrogen atom and refined in the same manner. The crystallographic details are listed in Supplementary Tables 1–3. The crystallographic data are deposited with the Cambridge Crystallographic Data Centre (CCDC), no. 1415661–1415671, 1415711–1415718 and 1415680–1415684.

### Thermal expansion

The thermal expansion coefficients were calculated from the lattice parameters obtained from variable temperature diffraction data collected from good single crystals of IMACET and its deuterated analogs (form I). The temperature was increased in 10 K increments from 270 K to 400–420 K, below the phase transition I → II. The axial thermal expansion coefficients along the principal axes were calculated by using the software PASCal^27^. The variable temperature unit cell parameters are listed in Supplementary Table 4.

### Thermal analysis

Differential Scanning Calorimetry (DSC) was carried out on TA DSC-Q2000 instrument in dynamic helium atmosphere (flow rate 50 mL min^−1^). Crystals were taken on a Tzero aluminium pan and heated from room temperature (300 K) to the corresponding phase transition temperature at rate 10 K min^−1^ (300–430 K). The cooling rate was reduced to 5 K min^−1^ in the 298–173 K range and to 2 K min^−1^ in the 173–103 K range.

### Computational methods

The calculations were performed using the CRYSTAL14 program^41^,^42^ and the B3LYP^43^ hybrid density functional. A Gaussian type orbital basis set was adopted of double zeta plus polarization quality^44^. The computational details are available as Supplementary Methods.

## Additional Information

**How to cite this article**: Panda, M. K. *et al.* Strong and Anomalous Thermal Expansion Precedes the Thermosalient Effect in Dynamic Molecular Crystals. *Sci. Rep.* **6**, 29610; doi: 10.1038/srep29610 (2016).

## Supplementary Material

Supplementary Information

Supplementary Movie 1

Supplementary Movie 2

Supplementary Movie 3

Supplementary Movie 4

Supplementary Movie 5

Supplementary Movie 6

Supplementary Movie 7

Supplementary Movie 8

Supplementary Movie 9

Supplementary Movie 10

Supplementary Movie 11

Supplementary Movie 12

Supplementary Movie 13

Supplementary Movie 14

Supplementary Movie 15

Supplementary Movie 16

Supplementary Movie 17

Supplementary Movie 18

## Figures and Tables

**Figure 1:**
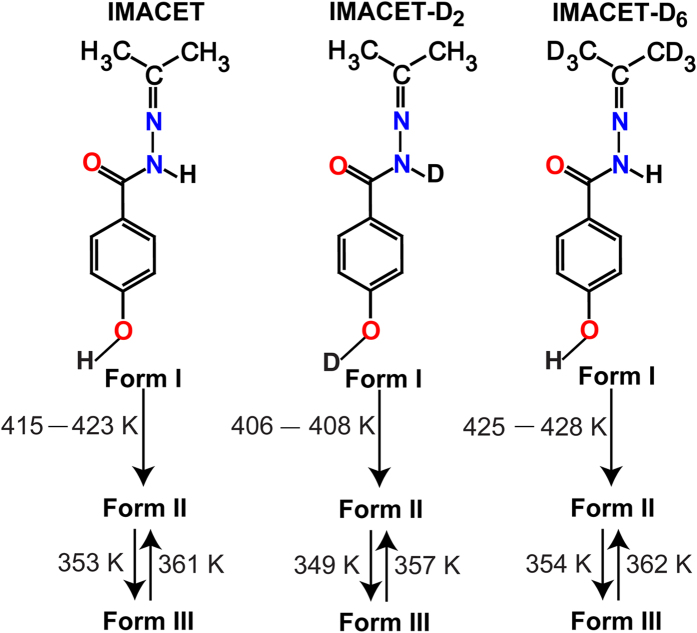
Chemical structures and phase transitions of IMACET and its deuterated analogues.

**Table 1:** Surface tension (*γ*) and relative area (*A*) of selected crystal faces of phases I, II and III.

Crystal face	Form					
	I		II		III	
	*γ*/(J m^−2^)	*A*/%	*γ*/(J m^−2^)	*A*/%	*γ*/(J m^−2^)	*A*/%
(001)	0.31	9.5	0.21	14.6	0.21	29.6
(100)	0.18	33.0	0.19	31.0	0.18	36.1
(101)	0.29	5.0	0.25	8.2	0.25	7.6
(010)	0.19	5.2	0.25	2.0	0.29	24.3
(011)	0.19	47.2	0.21	44.0	0.35	2.2
*z*/(*x*,*y*)^a^	1.67		0.95		0.78	

^a^z/(*x*,*y*) is the ratio of the *c* axis to the average of the *a* and *c* axes of the optimized cells.

## References

[b1] Wegst U. G. K., Bai H., Saiz E., Tomsia A. P. & Ritchie R. O. Bioinspired structural materials. Nature Mater. 14, 23–36 (2015). 25344782 10.1038/nmat4089

[b2] Dicker M. P. M., Rossiter J. M., Bond I. P. & Weaver P. M. Biomimetic photo-actuation: sensing, control and actuation in sun-tracking plants. Bioinspir. Biomim. 9, 036015 (2014). 24959885 10.1088/1748-3182/9/3/036015

[b3] Forterre Y., Skotheim J. M., Dumais J. & Mahadevan L. How the Venus flytrap snaps. Nature 433, 421–425 (2005). 15674293 10.1038/nature03185

[b4] Cully A., Clune J., Tarapore D. & Mouret J.-B. Robots that can adapt like animals. Nature 521, 503–516 (2015). 26017452 10.1038/nature14422

[b5] Li L., Weaver J. C. & Ortiz C. Hierarchical structural design for fracture resistance in the shell of the pteropod *Clio pyramidata*. Nat. Commun. 6, 6216, doi: 10.1038/ncomms7216 (2015). 25692262

[b6] Arndt E. M., Moore W., Lee W.-K. & Ortiz C. Mechanistic origins of bombardier beetle (Brachinini) explosion-induced defensive spray pulsation. Science 348, 563–567 (2015). 25931557 10.1126/science.1261166

[b7] van der Pijl L. Principles of dispersal in higher plants, Springer-Verlag: New York, 1982.

[b8] Roche E. T. *et al.* A bioinspired soft actuated material. Adv. Mater. 26, 1200–1206 (2014). 24227698 10.1002/adma.201304018

[b9] Sanchez C., Arribart H. & Guille M. M. G. Biomimetism and bioinspiration as tools for the design of innovative materials and systems. Nature Mater. 4, 277–288 (2005). 15875305 10.1038/nmat1339

[b10] He X. *et al.* Synthetic homeostatic materials with chemo-mechano-chemical self-regulation. Nature 487, 214–218 (2012). 22785318 10.1038/nature11223

[b11] Skoko Ž., Zamir S., Naumov P. & Bernstein J. The thermosalient phenomenon. “jumping crystals” and crystal chemistry of the anticholinergic agent oxitropium bromide. J. Am. Chem. Soc. 132, 14191–14202 (2010). 20860383 10.1021/ja105508b

[b12] Panda M. K. *et al.* Colossal positive and negative thermal expansion and thermosalient effect in a pentamorphic organometallic martensite. Nat. Commun. 5, 4811 (2014). 25185949 10.1038/ncomms5811

[b13] Naumov P., Sahoo S. C., Zakharov B. & Boldyreva E. Dynamic Single Crystals: Kinematic analysis of photoinduced crystal jumping (the photosalient effect). Angew. Chem. Int. Ed. 52, 9990–9995 (2013). 10.1002/anie.201303757 23873664

[b14] Nath N. K., Panda M. K., Sahoo S. C. & Naumov P. Thermally induced and photoinduced mechanical effects in molecular single crystals—a revival. CrystEngComm. 16, 1850–1858 (2014).

[b15] Sahoo S. C. *et al.* Kinematic and mechanical profile of the self-actuation of thermosalient crystal twins of 1,2,4,5-tetrabromobenzene: A molecular crystalline analogue of a bimetallic strip. J. Am. Chem. Soc. 135, 13843–13850 (2013). 23895677 10.1021/ja4056323

[b16] Panda M. K., Runčevski T., Husain A., Dinnebier R. E. & Naumov P. Perpetually self-propelling chiral single crystals. J. Am. Chem. Soc. 137, 1895–1902 (2015). 25581716 10.1021/ja5111927

[b17] Sahoo S. C., Panda M. K., Nath N. K. & Naumov P. Biomimetic crystalline actuators: structure–kinematic aspects of the self-actuation and motility of thermosalient crystals. J. Am. Chem. Soc. 135, 12241–12251 (2013). 23875700 10.1021/ja404192g

[b18] Centore R. *et al.* A series of compounds forming polar crystals and showing single-crystal-to-single-crystal transitions between polar phases. CrystEngComm. 14, 2645–2653 (2012).

[b19] Crocker A. G. & Bilby B. A. The crystallography of the martensite reaction in steel. Acta Metall. 9, 678–688 (1961).

[b20] Roitburd A. L. & Kurdjumov G. V. The nature of martensitic transformations. Mater. Sci. Eng. 39, 141–167 (1979).

[b21] Ubbelohde A. R. & Gallagher K. J. Acid-base effects in hydrogen bonds in crystals. Acta Crystallogr. 8, 71–83 (1955).

[b22] Mendel J., Mögel A. & Kolbe A. H/D Isotopic effect on the hydrogen bond between tertiary amine and alcohols. J. Mol. Liq. 29, 127–134 (1984).

[b23] Sobczyk L., Obrzud M. & Filarowski A. H/D isotope effects in hydrogen bonded systems. Molecules 18, 4467–4476 (2013). 23591926 10.3390/molecules18044467 PMC6269986

[b24] Keen D. A. *et al.* The hydrogen-bonding transition and isotope-dependent negative thermal expansion in H_3_Co(CN)_6_. J. Phys: Condens. Matter 22, 404202–404208 (2010). 21386563 10.1088/0953-8984/22/40/404202

[b25] Wulff G. Zur Frage der Geschwindigkeit des Wachsthums und der Auflösung der Krystallflächen. Z. Kristallog. 34, 449–530 (1901).

[b26] Causà M., Dovesi R. & Ricca F. Ab-initio Hartree-Fock investigation of the surface features of LiH slabs of different thickness. Surf. Sci. 237, 312–320 (1990).

[b27] Cliffe M. J. & Goodwin A. L. PASCal: a principal axis strain calculator for thermal expansion and compressibility determination. J. Appl. Crystallogr. 45, 1321–1329 (2012).

[b28] Goodwin A. L. *et al.* Colossal positive and negative thermal expansion in the framework material Ag_3_[Co(CN)_6_]. Science 319, 794–797 (2008). 18258911 10.1126/science.1151442

[b29] Goodwin A. L. & Kepert C. Negative thermal expansion and low-frequency modes in cyanide-bridged framework materials. J. Phys. Rev. B. 71, 140301-1–140301-4 (2005).

[b30] Yang C., Wang X. & Omary M. A. Crystallographic observation of dynamic gas adsorption sites and thermal expansion in a breathable fluorous metal–organic framework. Angew. Chem. Int. Ed. 48, 2500–2505 (2009). 10.1002/anie.200804739 19137517

[b31] Das D., Jacobs T. & Barbour L. J. Exceptionally large positive and negative anisotropic thermal expansion of an organic crystalline material. Nature Mater. 9, 36–39 (2010). 19935666 10.1038/nmat2583

[b32] Zhou H.-L., Zhang Y.-B., Zhang J.-P. & Chen X.-M. Supramolecular-jack-like guest in ultramicroporous crystal for exceptional thermal expansion behaviour. Nat. Commun. 6, 6917, doi: 10.1038/ncomms7917 (2015). 25898347 PMC4411299

[b33] Grobler I., Smith V. J., Bhatt P. M., Herbert S. A. & Barbour L. J. Tunable anisotropic thermal expansion of a porous Zinc(II) metal–organic framework. J. Am. Chem. Soc. 135, 6411–6414 (2013). 23581524 10.1021/ja401671p

[b34] Hunt S. J. *et al.* Flexibility transition and guest-driven reconstruction in a ferroelastic metal–organic framework. CrystEngComm 17, 361–369 (2015). 25632268 10.1039/c4ce01572j PMC4304274

[b35] Bruker AXS (Date of access: 15/10/2014). SAINT (version 7.34A). Madison, WI, USA. URL: https://www.bruker.com (2012).

[b36] Sheldrick G. M. (Date of access: 15/10/2014). SADABS. University of Göttingen, Göttingen, Germany. URL http://shelx.uni-ac.gwdg.de/SHELX/ (1996).

[b37] Sheldrick G. M. (Date of access: 15/10/2014). SHELXTL XT–Crystal Structure Solution (version 2014/4), Madison, WI, USA. URL http://shelx.uni-ac.gwdg.de/SHELX/(2010–2014).

[b38] Sheldrick G. M. SHELXT–Integrated space-group and crystal-structure determination. Acta Crystallogr. A 71, 3–8 (2015). 10.1107/S2053273314026370 PMC4283466 25537383

[b39] Sheldrick G. M. A short history of SHELX. Acta Crystallogr. A 64, 112–122 (2008). 18156677 10.1107/S0108767307043930

[b40] Sheldrick G. M. (Date of access: 15/10/2014). SHELXL2014. University of Göttingen, Göttingen, Germany. URL http://shelx.uni-ac.gwdg.de/SHELX/ (2014).

[b41] Dovesi R. *et al.* CRYSTAL14: A program for the ab initio investigation of crystalline solids. Int. J. Quantum Chem. 114, 1287–1317 (2014).

[b42] Dovesi R. *et al.* CRYSTAL14 User’s Manual, University of Torino, Torino, 2014, URL http://www.crystal.unito.it/index.php.

[b43] Becke A. D. Density-functional thermochemistry. IV. A new dynamical correlation functional and implications for exact-exchange mixing. J. Chem. Phys. 104, 1040–1046 (1996).

[b44] Francl M. M. *et al.* Self‐consistent molecular orbital methods. XXIII. A polarization‐type basis set for second‐row elements. J. Chem. Phys. 77, 3654–3665 (1982).

