# Supplementary material for: Strong and Anomalous Thermal Expansion Precedes the Thermosalient Effect in Dynamic Molecular Crystals
Source: Sci Rep. 2016 Jul 12;6:29610. doi: 10.1038/srep29610 (PMC4941691; doi:10.1038/srep29610)
Supplement: Supplementary Information [file srep29610-s1.pdf]

SUPPLEMENTARY MATERIAL

Strong and Anomalous Thermal Expansion Precedes the Thermosalient Effect in Dynamic Molecular Crystals

Manas K. Panda,[a] Roberto Centore,[b,*] Mauro Causà,[c] Angela Tuzi,[b] Fabio Borbone,[b] Panče Naumov[a,*]

[a]*New York University Abu Dhabi, PO Box 129188, Abu Dhabi, United Arab Emirates*
[b]*University of Naples Federico II, Department of Chemical Sciences, Via Cintia, I-80126, Naples, Italy*
[c]*University of Naples Federico II, Department of Chemical, Materials and Production Engineering, Piazzale V. Tecchio, I-80125, Naples, Italy*

Supplementary Methods

1. Synthesis and deuteration

Materials. 4-hydroxybenzohydrazide (CAS No. 5351-23-5) was obtained from Sigma Aldrich and used as received. HPLC grade acetone was purchased from VWR (UK) and used as received.

IMACET. The protiated (non-deuterated) form of IMACET was prepared as described in the literature.[1]

IMACET-D$_2$. In a typical preparation, 4-hydroxybenzohydrazide (0.360 g, 2.37 mmol), deuterated methanol (CH_3OD 99.8%, 7.5 mL) and D_2O (99.9%, 1 mL) were heated in a flask under a flow of nitrogen. Acetone (6 mL, 81.7 mmol) was added, and the mixture was refluxed 1 h under nitrogen. The solution was concentrated by gentle boiling in the air and cooled to room temperature in the closed flask. White solid precipitate separated out that was recovered by filtration and washed with D_2O. The product was recrystallized from a boiling saturated solution in CH_3OD by cooling to room temperature. The crystalline precipitate was recovered by filtration, washed with D_2O, dried 24 h at 60 °C and stored in a closed container connected to a $CaCl_2$ trap. Yield: 0.364 g (80%).

IMACET-D$_6$. In a typical preparation, a mixture of 4-hydroxybenzohydrazide (0.504 g, 3.31 mmol), methanol (20 mL) and water (1.5 mL) was heated in a flask with stirring. Deuterated acetone, CD_3COCD_3 (99.9%; 3 mL, 40.8 mmol) was added to the mixture, whereupon the slurry dissolved in several minutes. The solution was gently boiled for 1 h while adding deuterated acetone (9 mL) in small portions to retain a constant volume. Upon cooling to room temperature, white solid precipitate formed. The precipitate was filtered, washed with deuterated acetone, dried 24 h at 60 °C and stored in a closed container connected to a $CaCl_2$ trap. Yield: 0.558 g (85%).

Determination of the degree of deuteration. The degree of deuteration was determined from the ^1H NMR spectra recorded in D_6-DMSO. Because in the case of IMACET-D_2 there is a fast exchange between the deuterons of OD and ND and H_2O in D_6-DMSO, the determination of the degree of deuteration required quantitative evaluation of the change of the ratio H_2O/DMSO in the spectrum of the compound and comparison with the spectrum of the pure solvent. The same batch of solvent was used to dissolve the compound and to record the spectrum of the pure solvent. Representative spectra (of the product and the pure solvent) are shown as Supplementary Figures 1 and 2. The degree of deuteration was calculated to 99%. In the case of IMACET-D_6 there is no exchange between H_2O and the deuterated methyl groups, and the degree of deuteration was calculated from the spectrum of the compound alone. The degree of deuteration calculated from the spectra shown in Supplementary Figures 3 and 4 was 99 % in both cases.

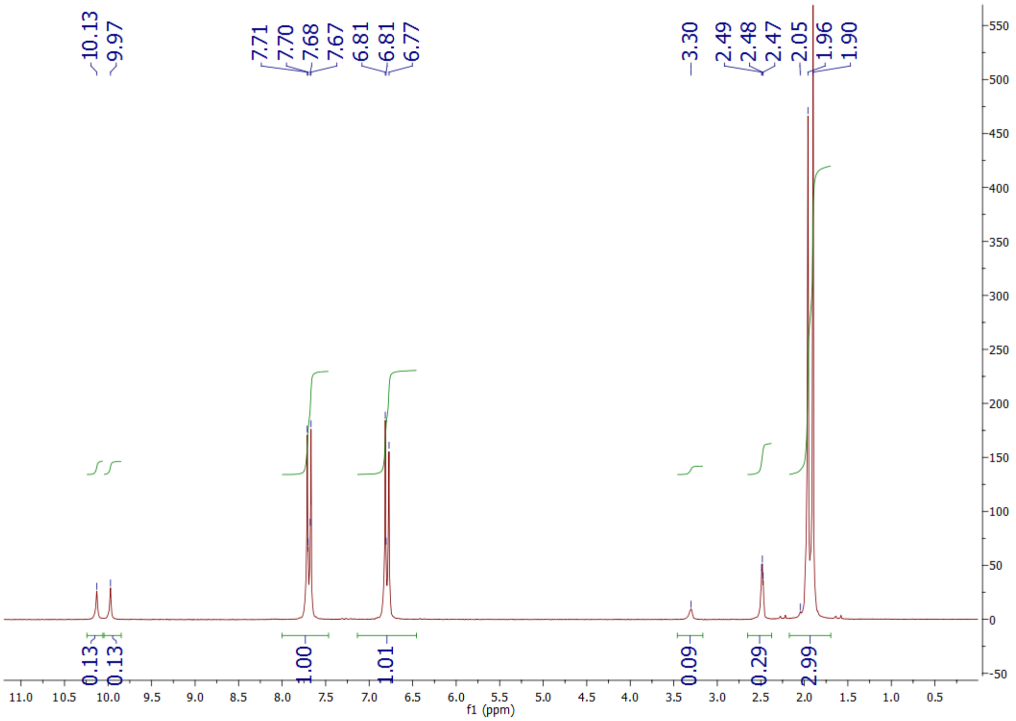

Supplementary Figure 1. ^{1}H NMR spectrum of IMACET-D_2 in D_6-DMSO (200 MHz).

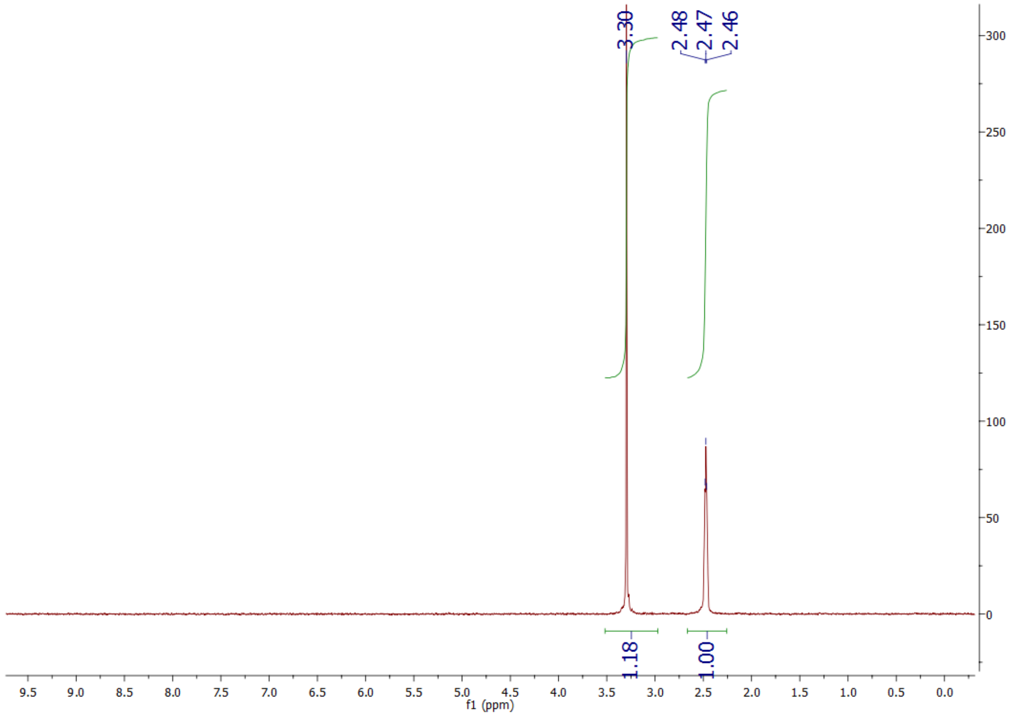

Supplementary Figure 2. ^{1}H NMR spectrum of the pure D_6-DMSO used as solvent to record the spectrum shown in Figure S1 (200 MHz).

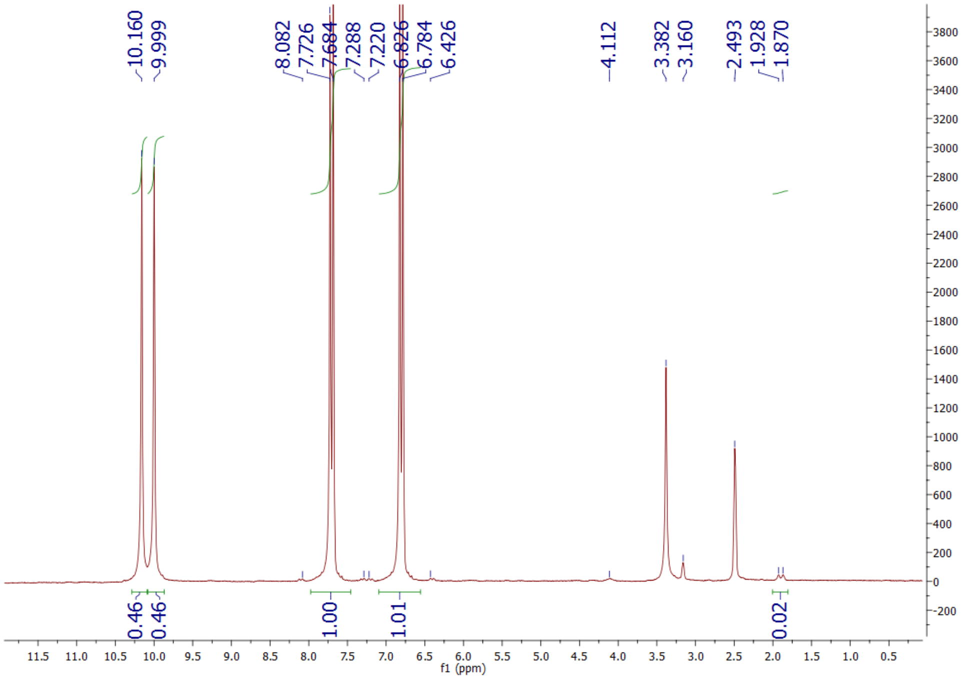

Supplementary Figure 3. ^{1}H NMR spectrum of IMACET-D_6 in D_6-DMSO (200 MHz).

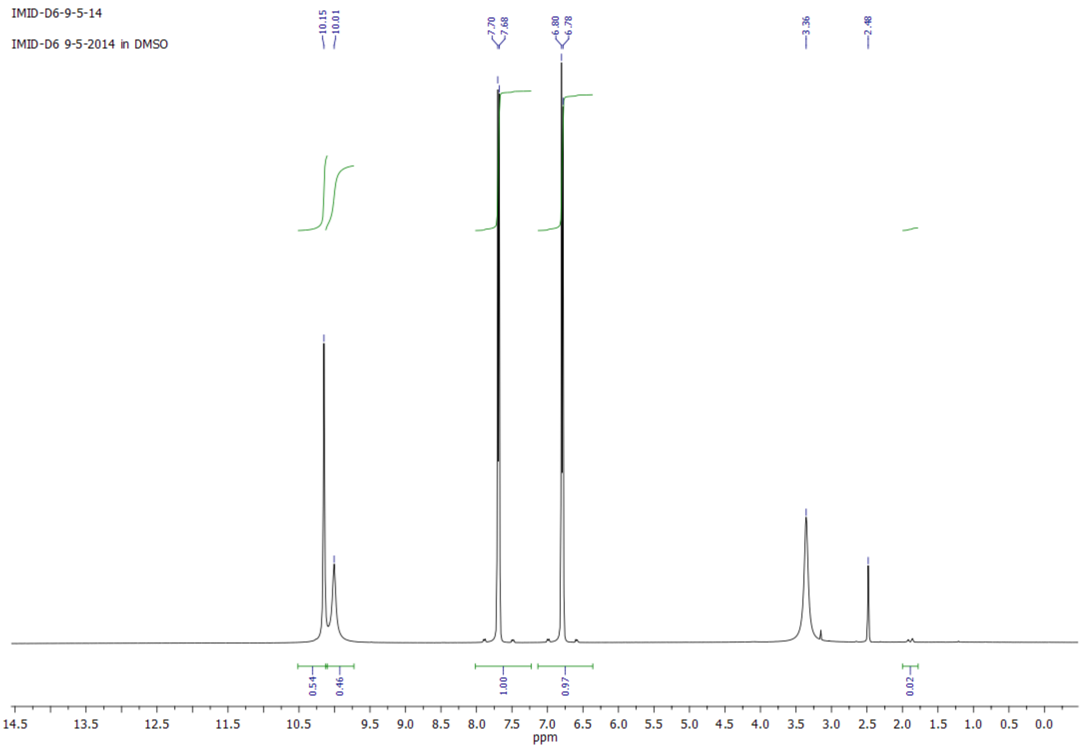

Supplementary Figure 4. ^{1}H NMR spectrum of IMACET-D_6 in D_6-DMSO (400 MHz).

2. Characterization

***Infrared spectroscopy*.** The infrared spectra were recorded to confirm the deuteration in the solid state samples and to assess any temperature effects on the structure on cooling from room temperature. The variable-temperature infrared spectra were recorded in KBr pellets using a Frontier MIR spectrometer (Perkin-Elmer) and an Optistat DN cryostat (Oxford Instruments) with quartz windows cooled with liquid nitrogen. The samples were kept in a static argon atmosphere and the spectra were collected at 10 K decrements from 298 K to 77 K. No significant changes that would indicate additional phase transitions at low temperature could be observed on cooling.

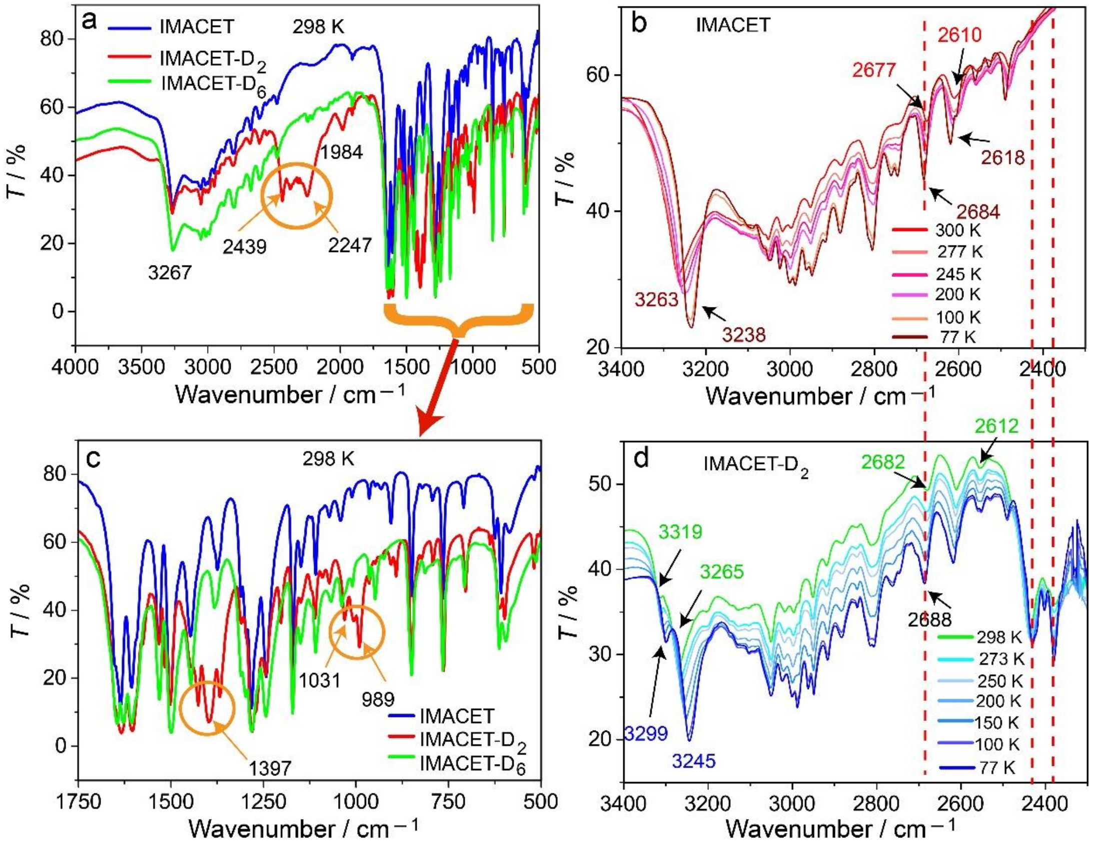

Supplementary Figure 5. Room temperature (a, c) and variable-temperature (b, d) IR spectra of IMACET and IMACET-D_2.

Differential Scanning Calorimetry. The DSC traces of IMACET and its deuterated analogues are shown in Supplementary Figure 6.

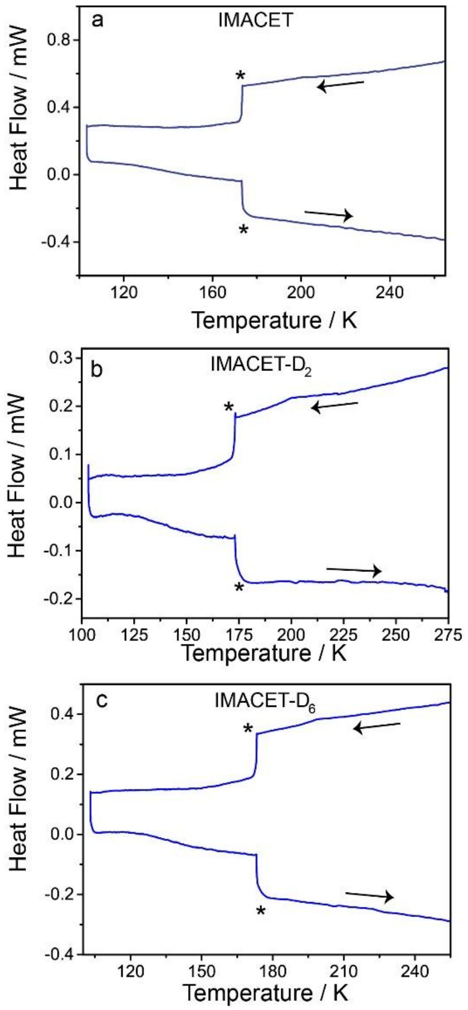

Supplementary Figure 6. Differential Scanning Calorimetry (DSC) curves of IMACET and its deuterated analogues recorded by cooling from room temperature to 103 K.

***Powder X-ray diffraction*.** In order to establish whether the thermally induced polymorphic behavior of IMACET single crystals occurs in the bulk material, powder diffraction patterns of IMACET and its deuterated analogs were recorded on PANalytical Empyrean diffractometer equipped with a sample heating stage. Intensity data were collected using Cu$K\alpha$ radiation (λ = 1.5418 Å) by 2Θ scans in the range 5–80°. The temperature was increased with a rate of 10 K min^{-1} and the data was collected at different temperatures: data for form I data was collected at 298 K, for form II at 420 K, and for form III data was collected after cooling of the heated sample to room temperature.

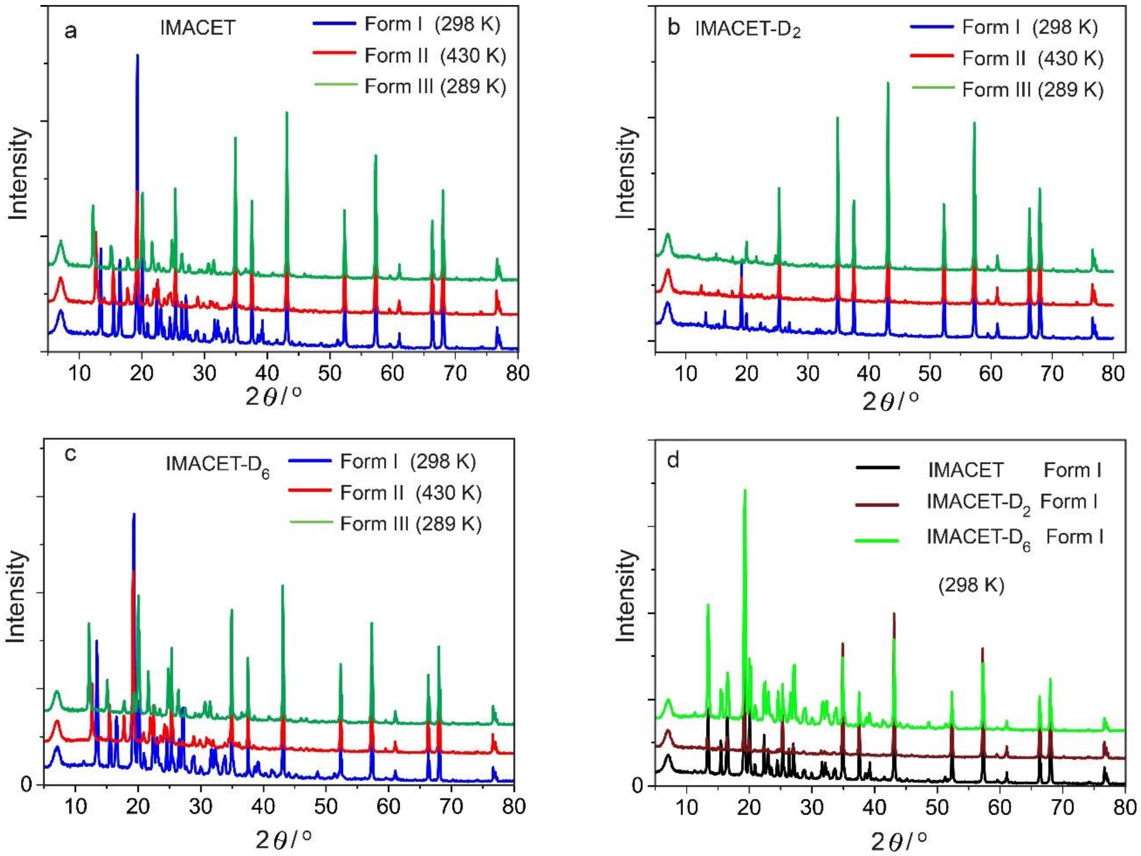

Supplementary Figure 7. Powder X-ray diffraction patterns of the three polymorphs of IMACET (a, b) and its deuterated analogues (b–d). The temperature at which the patterns were recorded is indicated in the plots. Panel d shows the diffraction patterns of form I of the protiated and deuterated analogues at 298 K. ***Note***: The samples for these measurements were prepared by very gentle grinding without applying excessive pressure to avoid suppression of the TS transition.

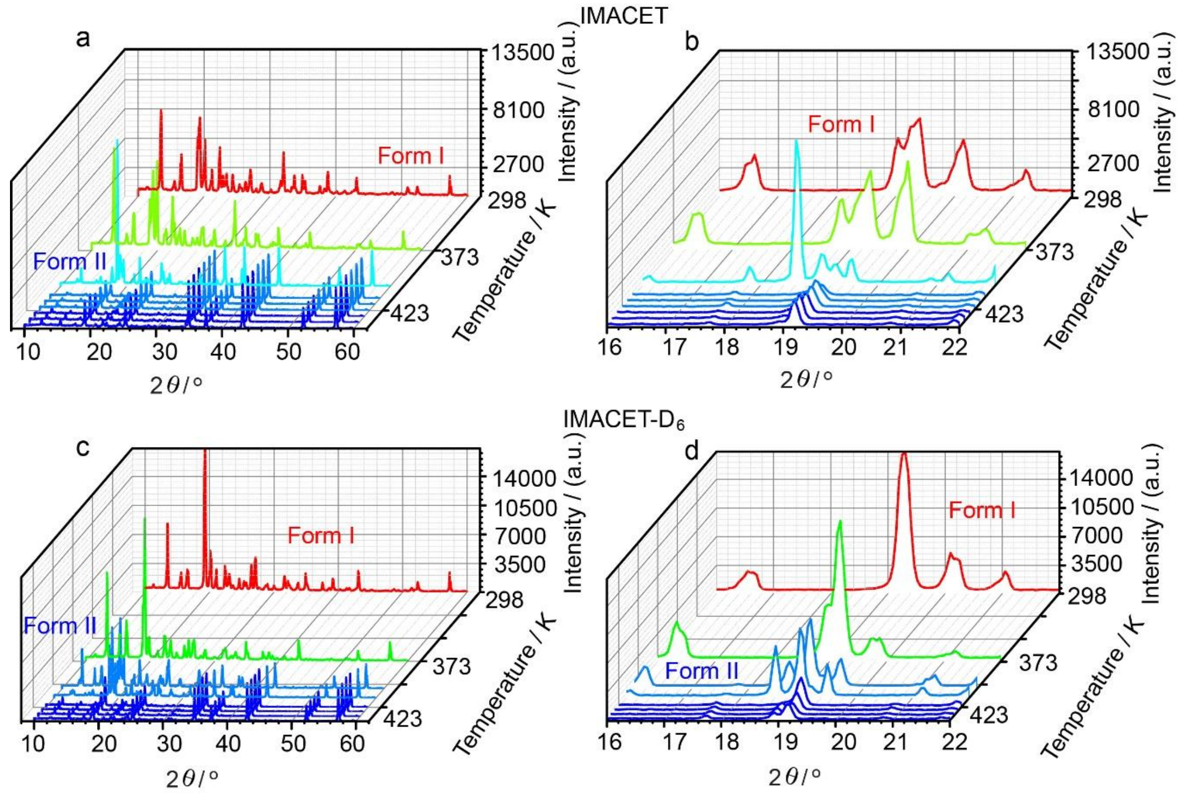

Supplementary Figure 8. Variable-temperature powder X-ray diffraction patterns of IMACET and IMACET-D_6. ***Note***: The samples for these measurements were prepared by very gentle grinding without applying excessive pressure to avoid suppression of the TS transition.

***Crystallization*.** In an attempt to obtain good crystals, IMACET-D_2 was recrystallized from EtOD at room temperature. Crystals that were obtained overnight were filtered, washed and collected. The crystal batch contained two habits: trapezoid-like crystals and oblique crystals that lacked sharp edges, corresponding, respectively, to phase III and phase I that crystallized concomitantly. The phase identity of the two forms was confirmed by their behavior on heating (phase transitions) observed with a microscope equipped with a hot stage. The crystal faces were indexed from single crystals by collecting a limited number of reflections with single crystal X-ray diffraction analysis. Typical crystal habits with indexed faces are shown in Supplementary Figure 9.

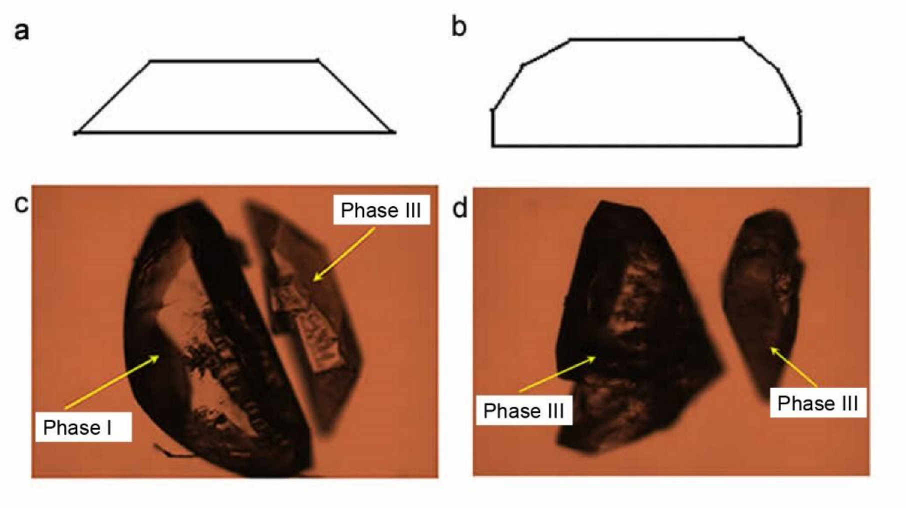

Supplementary Figure 9. Typical habits of IMACET-D_2 crystals. (a, b) 2D sketch of typical habits of crystals of IMACET-D_2 obtained by recrystallization. (c, d) Optical micrographs of typical crystal habits of concomitantly crystallized crystals of forms I and III of IMACET-D_2: well-developed crystals (c) and irregular crystals (d).

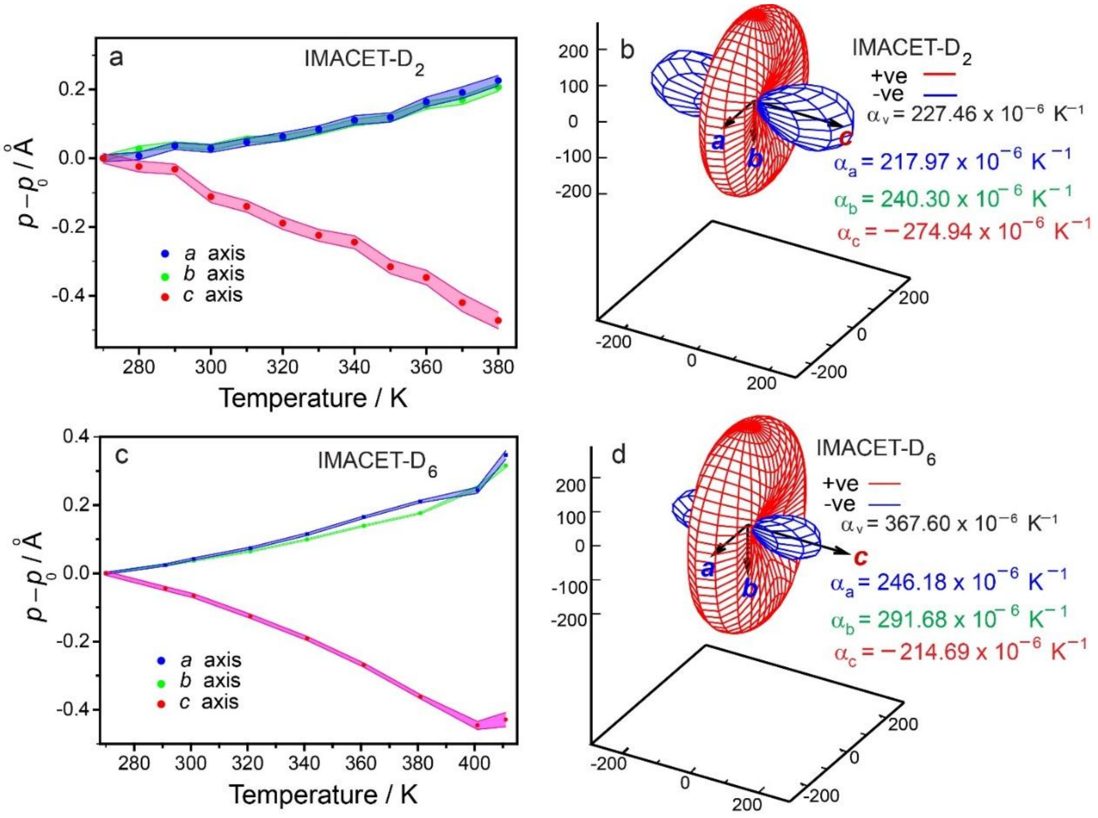

Supplementary Figure 10. Thermal expansion of unit cell parameters of Form I crystal of IMACET-D_2 (a,b) and IMACET-D_6 (c, d).

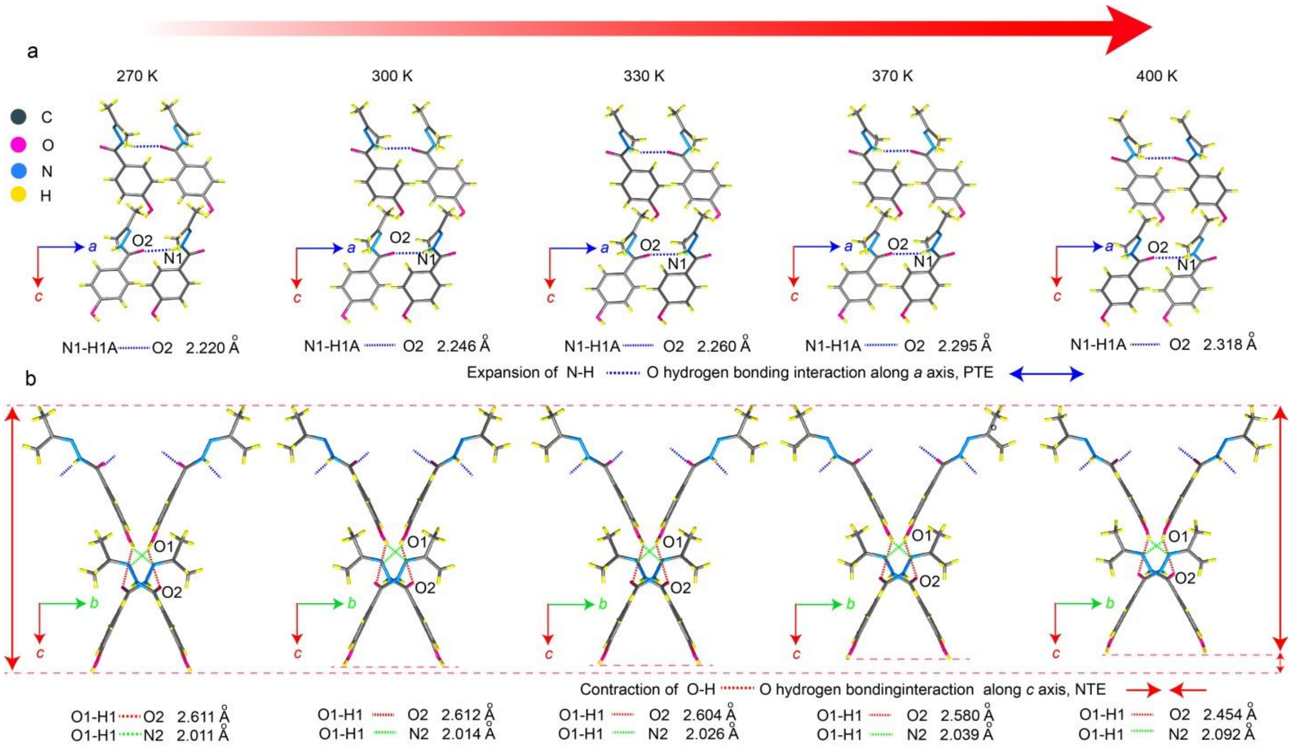

Supplementary Figure 11. Effect of temperature on different modes of hydrogen bonding in an IMACET crystal. Color codes: grey – carbon, red – oxygen, blue – nitrogen, yellow – hydrogen.

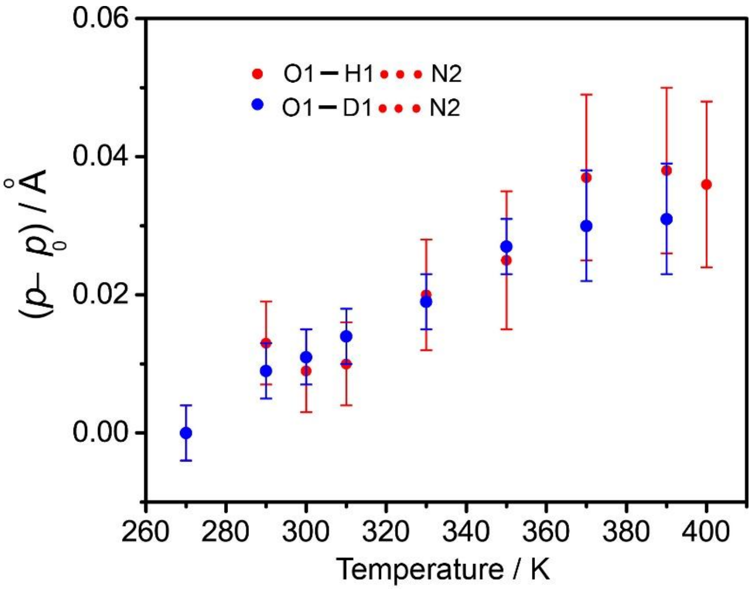

Supplementary Figure 12. Variations of the distance between the donor (D) and the acceptor (A) in the hydrogen bonds O1—H1···N2 in IMACET and O1—D1···N2 in IMACET-D_2 that account for anisotropy in the thermal expansion.

3. Computational details

All calculations were performed with the CRYSTAL14 package,[2,3] a periodic *ab initio* program based on atom-centered (Gaussian) basis sets. The geometry optimizations were performed within the framework of Density Functional Theory (DFT) making use of the hybrid B3LYP functional,[4] that includes part of the exact HF exchange, which reduces the self-interaction error and improves the performance in the description of the structure of solids. In all calculations, the positions of all atoms were fully relaxed along with the cell parameters. In the evaluation of the Coulomb and Hartree-Fock exchange series the five threshold parameters that determine the level of accuracy were set at 7, 7, 7, 7, 14. The threshold on the SCF energy was set to 10^{-8} Ha for the geometry optimizations, and 10^{-10} Ha for the frequency calculations. The reciprocal space was sampled according to a regular sub-lattice with shrinking factor equal to 4; this is absolutely reasonable due to the moderately large number of atoms in the unit cell that gives a physically small unit cell in the reciprocal space.

The dispersion forces that are absolutely relevant in the case of molecular crystals, were treated using London's method adapted to *ab initio* method by Grimme.[5,6] The DFT energy was corrected adding the term:

$$E_{disp} = s_6 \sum_{\mathbf{g}} \sum_{ij} f\left(R_{ij,g}\right) \frac{C_6^{ij}}{R_{ij,\mathbf{g}}^6}$$

where the summation is over all atom pairs *i,j* and lattice vectors **g** which define the cell, with exclusion of the $i = j$ contribution for $\mathbf{g} = 0$; C_6^{ij} is the dispersion coefficient for the *ij*-th pair of atoms; *f* is a damping function used to avoid near-singularities for small inter-atomic distances $R_{ij,\mathbf{g}}$; s_6 is a scaling factor that depends on the adopted DFT method (for B3LYP $s_6 = 1.0$), as estimated by fitting the binding energies of non-covalently bound complexes belonging to a large set of molecules. A double-ζ plus polarization quality basis set was used; the 6-31G(d,p)[7] is a small basis set in molecular calculations, however this does not hold for periodic band structure calculations which do not necessitate accurate description of the tails of the wavefunction due to the assistance from the basis function of the neighbor centers.

Supplementary Tables

Supplementary Table 1. Single crystal X-ray parameters of **IMACET** at different temperatures

	**Form I**				**Form II (363 K)**[1]	**Form III (293 K)**[1]
	**270 K**	**290 K**	**350 K**	**390 K**		
Chemical formula	$C_{10}H_{12}N_2O_2$	$C_{10}H_{12}N_2O_2$	$C_{10}H_{12}N_2O_2$	$C_{10}H_{12}N_2O_2$	$C_{10}H_{12}N_2O_2$	$C_{10}H_{12}N_2O_2$
M_r	192.22	192.22	192.22	192.22	192.2	192.2
Crystal system	Orthorhombic	Orthorhombic	Orthorhombic	Orthorhombic	Orthorhombic	Orthorhombic
Space group	$Pna2_1$	$Pna2_1$	$Pna2_1$	$Pna2_1$	$Pna2_1$	$Pna2_1$
a / Å	9.1415(8)	9.184(4)	9.281(3)	9.388(3)	9.968(5)	9.875(2)
b / Å	7.1872(7)	7.224(3)	7.308(2)	7.395(2)	8.082(5)	9.103(3)
c / Å	15.6583(15)	15.646(7)	15.397(5)	15.171(5)	13.267(8)	11.590(5)
$\alpha = \beta = \gamma$	90	90	90	90	90	90
V / Å^3	1028.78(17)	1038.0(8)	1044.3(5)	1053.2(6)	1069(1)	1041.8(6)
Z	4	4	4	4	4	4
ρ_{calc} / (g cm^{-3})	1.241	1.230	1.223	1.212	1.195	1.225
μ / mm^{-1}	0.088	0.087	0.087	0.086	0.085	0.087
F_{000}	408	408	408	408	408	408
θ_{min} / °	2.602	2.604	2.646	2.685	3.07	3.04
θ_{max} / °	28.379	28.390	28.397	28.404	24.99	27.49
Reflections collected	7714	7748	7800	7816	4875	5744
Independent reflections	2569	2585	2535	2505	1607	2305
GoF	1.031	1.055	1.077	1.009	1.121	1.055
Final R indices [$I > 2\sigma(I)$]	R_1 = 0.0330, wR_2 = 0.0852	R_1 = 0.0344, wR_2 = 0.862	R_1 = 0.0588, wR_2 = 0.1697	R_1 = 0.0699, wR_2 = 0.1947	R_1 = 0.0642, wR_2 = 0.1763	R_1 = 0.0424, wR_2 = 0.959
R indices (all data)	R_2 = 0.0398, wR_2 = 0.0899	R_2 = 0.0440, wR_2 = 0.930	R_2 = 0.0821, wR_2 = 0.1866	R_2 = 0.1072, wR_2 = 0.2213	R_2 = 0.974, wR_2 = 0.2047	R_2 = 0.636, wR_2 = 0.1073

[1]The values were taken from the Supplementary Reference 1.

Supplementary Table 2. Single crystal X-ray parameters for different polymorphs of **IMACET-D$_6$** at different temperatures

	Form I			Form II (383 K)	Form III (298 K)
	**100 K**	**200 K**	**298 K**		
Chemical formula	$C_{10}H_6D_6N_2O_2$	$C_{10}H_6D_6N_2O_2$	$C_{10}H_6D_6N_2O_2$	$C_{10}H_6D_6N_2O_2$	$C_{10}H_6D_6N_2O_2$
M_r	198.25	198.25	198.25	198.25	198.25
Crystal system	Orthorhombic	Orthorhombic	Orthorhombic	Orthorhombic	Orthorhombic
Space group	$Pna2_1$	$Pna2_1$	$Pna2_1$	$Pna2_1$	$Pna2_1$
a / Å	8.9425(4)	9.0367(2)	9.1643(5)	9.874(9)	9.9039(15)
b / Å	6.9773(2)	7.08380(10)	7.2011(3)	8.091(7)	9.1196(14)
c / Å	15.9432(5)	15.8066(3)	15.5857(6)	13.345(11)	11.6070(17)
$\alpha = \beta = \gamma$	90	90	90	90	90
V / Å^3	994.77(6)	1011.85(3)	1028.55(8)	1066(16)	1048.3(3)
Z	4	4	4	4	4
ρ_{calc} / (g cm^{-3})	1.324	1.301	1.280	1.235	1.256
μ / mm^{-1}	0.091	0.090	0.088	0.085	0.086
F_{000}	408	408	408	408	408
θ_{min} / °	3.187	3.151	3.116	2.944	2.841
θ_{max} / °	35.288	35.218	31.837	28.315	29.680
Reflections collected	8588	8869	7619	7711	8548
Independent reflections	3823	3933	3220	2369	2543
GoF	1.072	1.048	1.042	1.006	1.022
Final R indices [$I > 2\sigma(I)$]	R_1 = 0.0338, wR_2 = 0.0908	R_1 = 0.04154, wR_2 = 0.1087	R_1 = 0.0413, wR_2 = 0.1007	R_1 = 0.0563, wR_2 = 0.1658	R_1 = 0.0575, wR_2 = 0.1331
R indices (all data)	R_2 = 0.0386, wR_2 = 0.0943	R_2 = 0.0547, wR_2 = 0.1169	R_2 = 0.0643, wR_2 = 0.1124	R_2 = 0.1050, wR_2 = 0.1917	R_2 = 0.1058, wR_2 = 0.1547

Supplementary Table 3. Single crystal X-ray parameters for different polymorphs of **IMACET-D$_2$** at different temperatures

	**Form 1**			**Form II (373 K)**	**Form III (293 K)**
	**150 K**	**200 K**	**293 K**		
Chemical formula	$C_{10}H_{10}D_2N_2O_2$	$C_{10}H_{10}D_2N_2O_2$	$C_{10}H_{10}D_2N_2O_2$	$C_{10}H_{10}D_2N_2O_2$	$C_{10}H_{10}D_2N_2O_2$
M_r	194.23	194.23	194.23	194.23	194.23
Crystal system	Orthorhombic	Orthorhombic	Orthorhombic	Orthorhombic	Orthorhombic
Space group	$Pna2_1$	$Pna2_1$	$Pna2_1$	$Pna2_1$	$Pna2_1$
a / Å	8.9921(3)	9.0570(10)	9.1680(6)	9.900(3)	9.894 (4)
b / Å	7.0440(2)	7.1047(8)	7.2146(5)	8.134(3)	9.105 (3)
c / Å	15.8643(5)	15.8056(18)	15.5668(10)	13.270(4)	11.629 (4)
$\alpha = \beta = \gamma$	90	90	90	90	90
V / Å^3	1004.85(5)	1017.0(2)	1029.77(12)	1068.6(6)	1047.6 (6)
Z	4	4	4	4	4
ρ_{calc} / (g cm^{-3})	1.284	1.268	1.253	1.207	1.231
μ / mm^{-1}	0.090	0.089	0.088	0.085	0.087
F_{000}	408	408	408	408	408
θ_{min} / °	2.568	2.577	2.617	2.937	2.841
θ_{max} / °	34.778	28.431	28.212	23.935	28.267
Reflections collected	21088	7634	7463	5966	7433
Independent reflections	4146	2500	2472	2393	2538
GoF	1.054	1.015	1.144	0.1.054	1.089
Final R indices [$I > 2\sigma(I)$]	R_1 = 0.0363, wR_2 = 0.0991	R_1 = 0.0372, wR_2 = 0.0816	R_1 = 0.0537, wR_2 = 0.1029	R_1 = 0.0699, wR_2 = 0.2079	R_1 = 0.0886, wR_2 = 0.2276
R indices (all data)	R_2 = 0.0407, wR_2 = 0.1027	R_2 = 0.0489, wR_2 = 0.0880	R_2 = 0.0826, wR_2 = 0.1141	R_2 = 0.1099, wR_2 = 0.2083	R_2 = 0.1339, wR_2 = 0.2513

Supplementary Table 4. Temperature variation of the unit cell parameters (axes in Å, volume in Å^3) of **IMACET**, **IMACET-D$_2$** and **IMACET-D$_6$** (these values were used for calculate the thermal expansion coefficients in Figure 6)

T / K	*a* axis	e.s.d. (*a* axis)	*b* axis	e.s.d. (*b* axis)	*c* axis	e.s.d. (*c* axis)	Volume
**IMACET**							
270	9.1415	0.0008	7.1872	0.0007	15.6583	0.0015	1028.78
290	9.184	0.004	7.224	0.003	15.646	0.007	1038
310	9.2009	0.0017	7.2412	0.0014	15.552	0.003	1036.2
330	9.242	0.003	7.277	0.003	15.494	0.006	1042
350	9.281	0.003	7.308	0.002	15.397	0.005	1044.3
370	9.341	0.003	7.355	0.002	15.297	0.005	1050.9
390	9.388	0.003	7.395	0.002	15.171	0.005	1053.2
400	9.406	0.003	7.407	0.003	15.066	0.005	1049.6
**IMACET-D$_2$**							
270	9.153	0.005	7.201	0.004	15.678	0.007	1034.1
280	9.16	0.006	7.228	0.004	15.654	0.009	1036
290	9.189	0.005	7.241	0.004	15.646	0.008	1041.7
300	9.181	0.006	7.233	0.005	15.566	0.01	1034.7
310	9.2	0.006	7.256	0.005	15.538	0.01	1037
320	9.216	0.007	7.259	0.005	15.489	0.011	1036
330	9.237	0.006	7.279	0.005	15.454	0.01	1039
340	9.264	0.008	7.305	0.006	15.434	0.012	1045
350	9.272	0.008	7.317	0.007	15.362	0.013	1042
360	9.317	0.009	7.355	0.007	15.331	0.014	1050
370	9.344	0.012	7.371	0.009	15.258	0.019	1051
380	9.379	0.01	7.408	0.008	15.206	0.017	1057
**IMACET-D$_6$**							
270	9.1273	0.0017	7.1744	0.0011	15.65	0.003	1024.8
291	9.1517	0.0017	7.1979	0.0012	15.605	0.003	1027.9
301	9.1693	0.0016	7.2113	0.0011	15.584	0.003	1030.4
321	9.201	0.0017	7.2385	0.0011	15.524	0.003	1033.9
341	9.242	0.0018	7.2735	0.0012	15.459	0.003	1039.2
361	9.292	0.0017	7.3135	0.0012	15.381	0.003	1045.2
381	9.3378	0.0018	7.3506	0.0012	15.288	0.003	1049.4
401	9.371	0.007	7.425	0.005	15.204	0.009	1057.9
411	9.474	0.011	7.490	0.005	15.221	0.018	1080

Supplementary Table 5. Variable-temperature hydrogen bonding parameters for **IMACET** and **IMACET-D$_2$**

d(D•••A)/Å	Temperature / K								
	270	290	300	310	330	350	370	390	400
	IMACET								
O1—H1•••O2	3.159(2)	3.161(3)	3.150(2)	3.145(2)	3.141(4)	3.125(5)	3.114(5)	3.093(6)	3.087(6)
N1—H1A•••O2	3.040(2)	3.057(3)	3.060(2)	3.064(2)	3.076(4)	3.091(5)	3.110(5)	3.127(5)	3.131(6)
O1—H1•••N2	2.802(2)	2.815(3)	2.811(3)	2.812(3)	2.822(4)	2.827(5)	2.839(6)	2.840(6)	2.838(6)
	IMACET-D$_2$								
O1—D1•••O2	3.161(2)	3.157(2)	3.151(2)	3.148(2)	3.138(2)	3.125(2)	3.106(4)	3.079(4)	-
N1—D1A•••O2	3.045(2)	3.062(2)	3.068(2)	3.077(2)	3.092(2)	3.108(2)	3.115(3)	3.132(3)	-
O1—D1•••N2	2.805(2)	2.814(2)	2.816(2)	2.819(3)	2.824(2)	2.832(3)	2.835(3)	2.836(3)	

Supplementary References

[1] Centore, R., Jazbinsek, M., Tuzi, A., Roviello, A., Capobianco, A. & Peluso, A. A series of compounds forming polar crystals and showing single-crystal-to-single-crystal transitions between polar phases. *CrystEngComm* **14**, 2645−2653 (2012).

[2] Dovesi, R., Orlando, R., Erba, A., Zicovich-Wilson, C. M., Civalleri, B., Casassa, S., Maschio, L., Ferrabone, M., De La Pierre, M., D'Arco, P., Noel, Y., Causa, M., Rerat, M. & Kirtman, B. CRYSTAL14: A Program for the Ab Initio Investigation of Crystalline Solids. *Int. J. Quantum Chem.* **114**, 1287–1317 (2014).

[3] Dovesi, R., Saunders, V. R., Roetti, C., Orlando, R., Zicovich-Wilson, C. –M., Pascale, F., Civalleri, B., Doll, K.; Harrison, N. M., Bush, I. J., D'Arco, P., Llunell, M., Causà, M. & Noël, Y. *CRYSTAL14 User's Manual*, University of Torino, Torino, **2014**.

[4] Becke, A. D. Density–functional thermochemistry. IV. A new dynamical correlation functional and implications for exact–exchange mixing *J. Chem. Phys.* **104**, 1040–1046 (1996).

[5] Grimme, S. Accurate Description of van der Waals Complexes by Density Functional Theory Including Empirical Corrections. *J. Comp. Chem.* **25**, 1463–1473 (2004).

[6] Grimme, S. Semi-empirical GGA-Type Density Functional Constructed with a Long-Range Dispersion Correction. *J. Comp. Chem.* **27**, 1787–1799 (2006).

[7] Francl, M. M., Pietro, W. J., Hehre, W. J., Binkley, J. S., Gordon, M. S., DeFrees, D. J. & Pople, J. A. Self–consistent molecular orbital methods. XXIII. A polarization‐type basis set for second–row elements. *J. Chem. Phys.* **77**, 3654–3665 (1982).

Legends to the Supplementary Movies

Supplementary Movie 1. Hot-stage microscopy showing the thermosalient effect in IMACET form I crystal.

Supplementary Movie 2. Thermosalient actuation of IMACET form I crystals recorded from vertical (top) direction using microscope equipped with a high-speed camera (recording rate: 2000 s^{-1}).

Supplementary Movie 3. Thermosalient actuation of IMACET form I crystal, recorded from vertical (top) direction using microscope equipped with a high-speed camera (recording rate: 5000 s^{-1}).

Supplementary Movie 4. Splintering of IMACET form I crystal, recorded from lateral direction (recording rate: 1000 s^{-1}).

Supplementary Movie 5. Vertical jump of form I crystal of IMACET (recording rate: 1000 s^{-1}).

Supplementary Movie 6. Hot-stage microscopy showing the thermosalient effect in IMACET-D_2 form I crystal.

Supplementary Movie 7. Thermosalient effect of IMACET-D_2 form I crystal (recording rate: 5000 s^{-1}).

Supplementary Movie 8. Thermosalient and non-thermosalient transitions of form I and form III crystals of IMACET-D_2.

Supplementary Movie 9. Hot-stage microscopy showing thermosalient transition of IMACET-D_2 form I crystal and non-thermosalient transition of form III crystal.

Supplementary Movie 10. Hot-stage microscopy showing the thermosalient effect of IMACET-D_6 form I crystal.

Supplementary Movie 11. Cold-stage (liquid N_2) microscopy of IMACET form I crystal, showing absence of thermosalient effect at low temperature.

Supplementary Movie 12. Cold-stage (liquid N_2) microscopy of IMACET-D_2 form I crystal, showing absence of thermosalient effect at low temperature.

Supplementary Movie 13. Cold-stage (liquid N_2) microscopy of IMACET-D_6 showing absence of thermosalient effect in IMACET-D_6 form I crystal.

Supplementary Movie 14. Actuation of form I crystals of IMACET, IMACET-D_2 and IMACET-D_6 (Note that because of limitation with memory that limits the recording time with

high-speed camera, the movies were recorded in fragments and combined together; recording rate: 2000 s^{-1}).

Supplementary Movie 15. Comparison of the thermosalient behavior of form I crystals of IMACET-D_2 and IMACET.

Supplementary Movie 16. Comparison of the thermosalient behavior of form I crystals of IMACET-D_6 and IMACET.

Supplementary Movie 17. Structural changes, based on X-ray diffraction data, showing the thermal expansion prior to the phase transition and the mechanism of the phase transition that accounts for the thermosalient effect in IMACET.

Supplementary Movie 18. Reversible macroscopically observable volume change (“breathing”) during reversible non-thermosalient transition of IMACET-D_2 crystals (recording rate: 2000 s^{-1}; for enhanced visualization, the video is shown at 20× the original rate).
